# Systems Biology-Based Analysis Indicates Global Transcriptional Impairment in Lead-Treated Human Neural Progenitor Cells

**DOI:** 10.3389/fgene.2019.00791

**Published:** 2019-09-10

**Authors:** Clovis F. Reis, Iara D. de Souza, Diego A. A. Morais, Raffael A. C. Oliveira, Danilo O. Imparato, Rita M. C. de Almeida, Rodrigo J. S. Dalmolin

**Affiliations:** ^1^Bioinformatics Multidisciplinary Environment — IMD, Federal University of Rio Grande do Norte, Natal, Brazil; ^2^Institute of Physics and National Institute of Science and Technology: Complex Systems, Federal University of Rio Grande do Sul, Porto Alegre, Brazil; ^3^Department of Biochemistry — CB, Federal University of Rio Grande do Norte, Natal, Brazil

**Keywords:** lead exposure, lead poisoning, transcriptogramer, RNA-seq, transcriptome analysis, network inference, data integration, network visualization

## Abstract

Lead poisoning effects are wide and include nervous system impairment, peculiarly during development, leading to neural damage. Lead interaction with calcium and zinc-containing metalloproteins broadly affects cellular metabolism since these proteins are related to intracellular ion balance, activation of signaling transduction cascades, and gene expression regulation. In spite of lead being recognized as a neurotoxin, there are gaps in knowledge about the global effect of lead in modulating the transcription of entire cellular systems in neural cells. In order to investigate the effects of lead poisoning in a systemic perspective, we applied the transcriptogram methodology in an RNA-seq dataset of human embryonic-derived neural progenitor cells (ES-NP cells) treated with 30 µM lead acetate for 26 days. We observed early downregulation of several cellular systems involved with cell differentiation, such as cytoskeleton organization, RNA, and protein biosynthesis. The downregulated cellular systems presented big and tightly connected networks. For long treatment times (12 to 26 days), it was possible to observe a massive impairment in cell transcription profile. Taking the enriched terms together, we observed interference in all layers of gene expression regulation, from chromatin remodeling to vesicle transport. Considering that ES-NP cells are progenitor cells that can originate other neural cell types, our results suggest that lead-induced gene expression disturbance might impair cells’ ability to differentiate, therefore influencing ES-NP cells’ fate.

## Introduction

Lead is largely used in industry and is very toxic to biological systems. This heavy metal accumulates in hard tissues, remaining in bones and teeth for decades (Ronis et al., 2001). Lead systemic effects can be observed through a wide range of lead poisoning symptoms. It includes anemia, abdominal pain, vomiting, cardiovascular system impairment, nephropathies, and abnormal spermatogenesis (Flora et al., 2012; Mitra et al., 2017). Several studies describe lead toxicity in central nervous system as well as its relation to irreversible brain development impairment (Finkelstein et al., 1998; Baranowska-Bosiacka et al., 2012; Stansfield et al., 2012). This diversity of systemic effects indicates that lead interacts with many cellular components. A recent review conducted by our group highlighted proteins that are described to directly interact with lead and how those interactions could be related to lead poisoning symptoms (de Souza et al., 2018). Lead strongly inhibits NMDA receptors, a transmembrane calcium channel from glutamatergic neuronal pathways that performs a crucial role in brain development (Nihei et al., 2000; Baranowska-Bosiacka et al., 2012). NMDA receptor inhibition is related to lead binding in the NMDA receptor zinc-binding site. In fact, much of lead toxicity relies on its binding to metalloproteins with divalent ions as prosthetic groups, especially calcium and zinc. Metalloproteins are involved with a variety of cellular processes, from regulation of cellular ion balance to activation of signaling cascades, inducing changes in cell metabolism. Calcium-interacting proteins perform important roles in cell transduction cascades. Lead mimics calcium on protein kinase C (PKC) isoforms and calmodulin (CaM), triggering their downstream signaling cascades (Richardt et al., 1986; Sun et al., 1999). Lead interacts with zinc finger transcription factor family members by substituting zinc ions, impairing zinc fingers DNA binding, and directly interfering in gene expression (Zawia et al., 1998; Reddy and Zawia, 2000; Ghering et al., 2005). Taken together, lead interaction to metalloproteins can massively interfere at the cell transcription profile (Guilarte and McGlothan, 1998; Ordemann and Austin, 2016).

Here, we investigate lead poisoning transcriptional effects from a systemic perspective by applying the transcriptogram pipeline to a lead poisoning experiment data publicly available. Transcriptogram pipeline consists of a systems biology-based methodology designed to perform transcriptional analysis of functionally associated gene groups in case-control experiments (Rybarczyk-Filho et al., 2011). It implements a non-supervised approach based on protein–protein interaction (PPI) to identify differentially expressed protein subnetworks. Transcriptogram pipeline results in global transcriptional profiles of each biological phenomenon evaluated, evidencing the biochemical systems collectively altered in consequence of a given disturbance. We analyzed an RNA-seq experiment of human embryonic-derived neural progenitor cells (ES-NP cells) treated with 30 μM lead acetate for 1 to 26 days (Jiang et al., 2017). Our analysis has focused on identifying early and late global lead-induced transcriptional alterations. Supporting previous findings from Jiang et al. (2017), our results showed that lead interferes in many cellular processes (e.g., cell cycle regulation, macromolecule metabolism, response to DNA damage, and cytoskeleton organization). Additionally, our analysis pointed to different levels of lead-induced perturbation when considering early and late exposure. It was possible to observe a high transcriptional downregulation of well-connected systems at initial days of exposure. In a second time interval, we observed an overall transcriptional modification suggesting that lead exposure results in massive interference in gene expression regulation in ES-NP cells.

## Materials and Methods

### Data Selection and Preprocessing

Data from 26 RNA-seq of human embryonic-derived neural progenitor cells (ES-NP cells) samples treated with lead acetate 30 μM in a time-series experiment (day 1 to day 26) and 27 respective control samples (day 0 to day 26) were obtained from Gene Expression Omnibus (accession GSE84712) (Jiang et al., 2017). Raw data sequence reads were downloaded from Sequence Read Archive (accession SRP079342) and processed following new Tuxedo protocol (Pertea et al., 2016) using Ensembl GRCh38 Human genome reference and annotation (release 91). Counts were then filtered, normalized, and converted into log2-counts-per-million (log-CPM) following *limma* R/Bioconductor package protocol (Ritchie et al., 2015). Principal component analysis (PCA) was performed with all samples in order to check data quality (**Supplementary Figure S1**).

### Group Selection and Transcriptogram Analysis

Differential expression of functionally associated gene groups among ES-NP cells treated with lead acetate 30 μM and respective controls was performed using *transcriptogramer* R package (Morais et al., 2019). The *transcriptogramer* is a systems biology-based method to analyze transcriptomes, which uses PPI information to identify differentially expressed functionally associated gene groups in case-control designed experiments (Rybarczyk-Filho et al., 2011; Morais et al., 2019). Briefly, the method sorts the genes in one dimension by the probability that their products collaborate in a biological function and uses the resulting ordered gene list to project the expression of functionally associated genes in a sliding window with a given radius. As a result, the *transcriptogramer* evaluates the expression of entire genetic/biochemical systems in a biological condition. *Transcriptogramer* package uses the *limma* algorithm to compute the significance of functionally associated gene groups expression variation in case-control experiments, identifying differentially expressed clusters in a non-supervised way.

To allow *transcriptogramer* statistical evaluation, lead-treated samples were grouped by expression similarity using PCA clustering. Clustering analysis was conducted on the 30 μM lead-treated samples using the first 17 principal components, which corresponds to about 95% of the cumulative variance (**Supplementary Figure S2**). Group selection was performed by a non-supervised algorithm using *HCPC* function from *FactoMineR* R/CRAN package (Lê et al., 2008) and methodology from Husson et al. (2010). Clustering was carried out using automatic cluster detection, identifying three groups of samples. Group 1, defined as time-interval 0, included only two samples (day 1 and 2) and therefore was discarded for transcriptogramer analysis. The remaining two groups were defined for the study as time-interval 1 (days 3 to 11; n = 9) and time-interval 2 (days 12 to 26; n = 15) (**Supplementary Figure S3**).

### Progression of the Neuronal Markers

To characterize the differentiation of the neural progenitors (NPCs) to neuronal cells, we evaluated the expression (log-CPM) of a set of well-known markers for NPCs and neurons. We used the genes *MSI1*, *NES*, *NOTCH1*, and *SOX1* as NPC markers, and genes *TH*, *NEUROD6*, *DCX*, *RBFOX3*, *GAD1*, and *GAD2* as neuronal markers (Kaneko et al., 2000; Lutolf et al., 2002; Wiese et al., 2004; Cossette et al., 2005; Suter et al., 2009; White and Thomas, 2012; Gusel’nikova and Korzhevskiy, 2015; Khan et al., 2017; Mathews et al., 2017). As explained in the section above, the transcriptional data used here are a time-series experiment including 53 samples: 26 lead-treated (lead acetate 30 μM) samples (day 1 to day 26), 26 control samples (day 1 to day 26), and day 0 sample. To test marker expression variation by time, we subdivide both control and treated samples into three groups each: i) time-interval 0 (from day 0 to 2), ii) time-interval 1 (from day 3 to 11), and iii) time-interval 2 (from day 12 to 26). The same unique day 0 sample was used to compose control time-interval 0 and treated time-interval 0. The marker expression differences among the time intervals (for both control and lead-treated) were obtained by a pairwise t-test using the pairwise.t.test function from stats R package. The p-values were corrected by the FDR method, and p-values below 0.01 are considered significant.

### Transcriptogramer Analysis

*Transcriptogramer* differential expression method was applied to each time interval comparing treated groups with the respective control, using ordered gene list built under STRING V10 combined score ≥700, window radius = 80. Differential expression statistical relevance was assessed by Fisher test with Benjamini-Hochberg adjustment of p-values (p-value ≤ 0.001). After differential expression analysis, the PPI networks of significant clusters were plotted using *clusterVisualization transcriptogramer* function, and GO enrichment analysis of each differentially expressed cluster was performed using *clusterEnrichment* function. Final network manipulation was performed using *RCytoscape* R/Bioconductor (Shannon et al., 2013) and *RedeR* R/Bioconductor packages (Castro et al., 2012). Connectivity graphs (**Figures 2B, D**) represent the average network connectivity and were plotted using the *circlize* R/CRAN package (Gu et al., 2014).

### GO Terms Hierarchical Networks

The hierarchical networks of significantly enriched GO terms in each differentially expressed cluster were built based on the similarity among any pair of enriched GO terms (i.e., the number of shared genes in each pair of GO terms). The method calculates the Jaccard’s Index of each GO term pair, all-against-all, and builds an adjacency matrix that is used to compute a dendrogram. The resulting dendrogram is then plotted as a tree-and-leaf network using *RTN* R/Bioconductor package (Fletcher et al., 2013; Castro et al., 2016). Each node in the resulting hierarchical network represents a significantly enriched GO term. Node size is proportional to GO term size (i.e., the number of genes associated to the GO term), and the node color represents the ratio of significantly enriched genes over the count of all annotated GO term genes, normalized by the max ratio found in the cluster. Clusters dendrograms were generated using 0.25 Jaccard as a cutoff parameter, and the final layout was shown using *RedeR* R/Bioconductor package (Castro et al., 2012).

## Results

### Expression Markers for ES-NP Cells Differentiation

**Figure 1:**
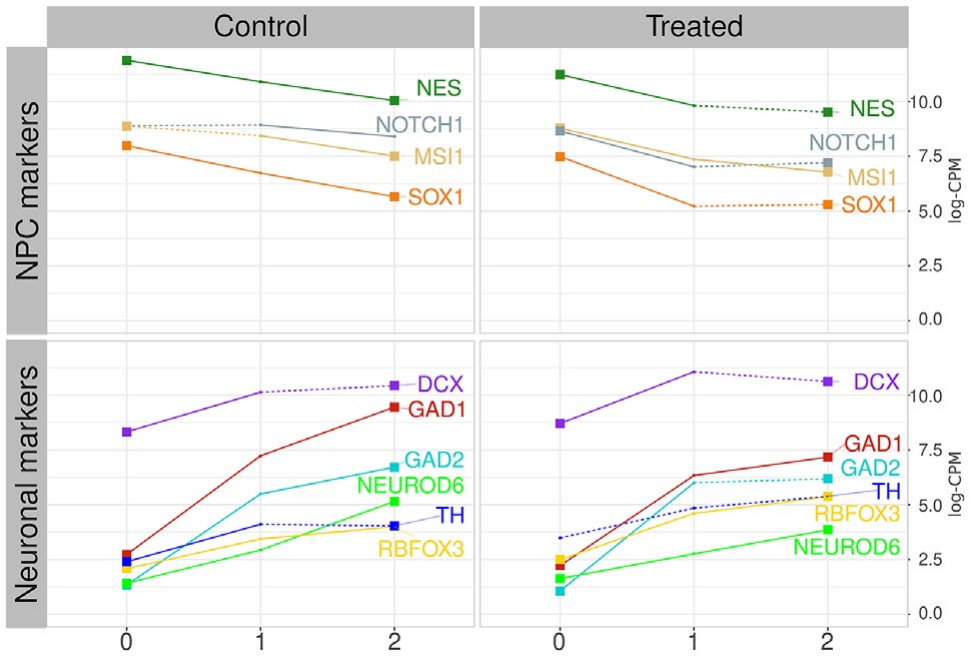
NPC and neuronal marker progression. Left graphics show the control group average expression values in log-CPM, for NPC markers (on top) and neuronal markers (on bottom). Right graphics show the lead-treated sample average expression values. Colors represent distinct markers. Numbers at the X-axis identify the time intervals starting on zero, for samples from day 0 to 2, and time-intervals 1 and 2, from day 3 to 11, and day 12 to 26, respectively. Numbers at the Y-axis represent the averaged value of samples expression inside each time interval. Continuous lines represent significant alterations on the expression level when compared with the previous time interval (pairwise t-test, corrected by FDR with p-value ≤ 0.01). Dotted lines represent alterations considered non-significant. Lines started and finished with a squared dot represent markers that present significant changes detected by pairwise t-test between time-interval zero and time-interval 2.

ES-NP cells are neural progenitor cells able to differentiate into neurons and glial cells (Selvaraj et al., 2012) and the modified protocol used by Jiang and collaborators points to neuronal differentiation (Chambers et al., 2009; Jiang et al., 2017). The samples comprise a time-course experiment from 26 RNA-seq of human embryonic-derived neural progenitor cells (ES-NP cells) samples treated with lead acetate 30 μM (day 1 to day 26) and 26 respective control samples (day 1 to day 26) plus day 0. The treated samples were grouped by PCA followed by non-supervised clustering, producing three groups of samples: i) time-interval 0, comprising days 0 to 2; ii) time-interval 1, comprising days 3 to 11; and iii) time-interval 2, comprising days 12 to 26 (see *Materials and Methods* and Supplementary Material Online for details). We evaluated the expression dynamics of neuroprogenitor cells (NPCs) marker genes, as well as neuronal cell marker genes, to evaluate the ES-NP cells differentiation progression (**Figure 1**, **Supplementary Figures S4** and **S5**). **Figure 1** shows the expression of NPC markers (*NES*, *NOTCH1*, *MSI1*, and *SOX1*) and neuronal cell markers (*DCX*, *GAD1*, *GAD2*, *NEUROD6*, *TH*, and *RBFOX3*) in both control cells and treated cells. *NES* and *MSI1* are described as highly expressed in progenitor-like cells (Kaneko et al., 2000; Wiese et al., 2004). *NOTCH1* and *SOX1* are also described as highly expressed in NPCs and are the main drivers of differentiation process (Lutolf et al., 2002; Suter et al., 2009), while *DCX* is an important marker for immature neurons (Mathews et al., 2017). *TH* and *NEUROD6* are recognized as dopaminergic markers (White and Thomas, 2012; Khan et al., 2017). *GAD1* and *GAD2* are gabaergic differentiation markers and were found to have a good correlation with *TH* expression in gabaergic neurons (Cossette et al., 2005). *RBFOX3* is a postmitotic neuronal marker (Gusel’nikova and Korzhevskiy, 2015). **Figure 1** shows the mean expression levels of the aforementioned genes in each time interval for control and treated cells. The majority of NPC markers decreased their expression in both control and lead-treated samples when comparing the initial and final time intervals. The exception is *NOTCH1* expression, which was significantly altered in control samples only when comparing time-interval 1 and 2, but not when comparing time-interval 0 and 2. Neuronal marker expression increased in the same period in both control and lead-treated samples, except for *TH* expression, which was not significantly altered in lead-treated samples (**Figure 1**). The dynamics of NPC and neuronal marker expression observed in **Figure 1** is consistent with neuronal differentiation since NPC marker expression decreased while neuron marker expression increased along with the experiment. However, the markers transcriptional dynamics are significantly different when comparing treated and control samples (**Supplementary Figures S4** and **S5**).

### Transcriptional Profile and Clusters Network Dynamics on Time Intervals

To evaluate the transcriptional disturbance caused by lead, we applied the transcriptogram pipeline to time-interval 1 and 2, always comparing lead-treated samples to their respective control samples (see *Materials and Methods* and Supplementary Material Online for details). The transcriptogram is a systems biology-based method for transcriptional analysis, which uses PPI information to build an ordered gene list where interacting genes are expected to get closer to each other (Rybarczyk-Filho et al., 2011). The ordered gene list is then used to project the expression of functionally associated gene groups that are likely to participate in common biochemical pathways.

**Figure 2:**
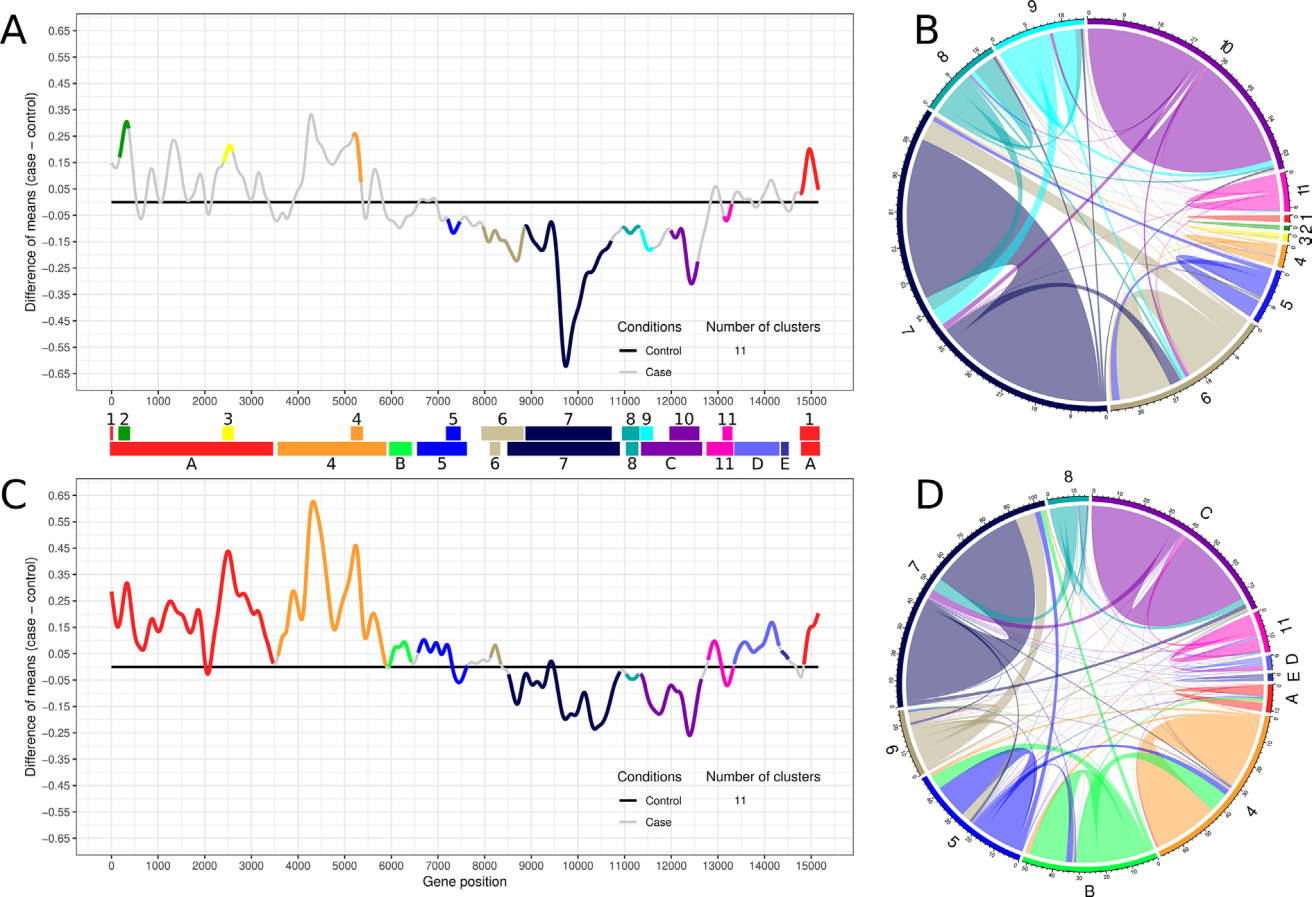
Transcriptogram and connectivity diagram of time-intervals 1 and 2. **(A, C)** The transcriptogram of time-interval 1 (day 3 to 11, n = 9) and time-interval 2 (day 12 to 26, n = 15), respectively, from control and treated samples. The X-axis represents gene position, and the Y-axis the relative expression. The solid black line represents control expression average, and the solid gray line represents the lead treatment expression average relative to control. Colored lines highlight the groups of differentially expressed genes that compose the 11 identified clusters. Both transcriptograms were performed using radius = 80, and differentially expressed clusters were selected with p-value ≤ 0.001. Clusters are represented as PPI networks and were functionally characterized by GO biological process enrichment. Colored bars between transcriptograms illustrate the area occupied by each cluster on the X-axis. Top bar indicates the time-interval 1 clusters, sequentially enumerated from left to right. Bottom bar indicates the time-interval 2 clusters identified by the correspondent former cluster number and color. Clusters that arise only on time-interval 2 or embrace two or more clusters from time-interval 1 are labeled with capital letters and identified with a new color or the same color of the former cluster with greater overlapping area, respectively. **(B, D)** The average network connectivity from time-interval 1 differentially expressed clusters. The area occupied by each cluster in the circumference is proportional to the cluster average connectivity in the subnetwork (ratio between the number of cluster connections and the number of cluster nodes). Clusters are identified by the same labels and colors used in **(A)** and **(C)**. Colored lines on the circle perimeter represent the area occupied by each cluster, normalized by the total number of cluster component interactions. Colored lines inside the circle illustrate the inner and outer average connectivity, defined as the ratio between the number of all cluster protein connections and the number of proteins of each cluster. Lines connecting the same cluster represent inner connectivity (connections among genes belonging to the same cluster), while lines connecting different clusters represent outer connectivity. Node connections not included at any clusters are not represented.

*Transcriptogramer* analysis of time-interval 1 identified 11 clusters of differentially expressed gene sets, which were sequentially numbered (**Figure 2A**). Clusters 1 to 4 were upregulated while clusters 5 to 11 were downregulated (**Figure 2A**). **Figure 2B** represents the subnetwork involving the 11 differentially expressed clusters of time-interval 1. All clusters have more inner than outer connections, indicating that methodology was able to identify functionally associated gene groups. This representation illustrates how each cluster contributes proportionally to the time-interval 1 interactome (**Supplementary Figure S18**). Collectively, downregulated clusters are more connected when compared to upregulated clusters on time-interval 1 (**Figure 2B**).

*Transcriptogramer* analysis of time-interval 2 shows a global alteration in cell transcription pattern, suggesting a progressive cellular response among time intervals. As shown in **Figure 2C**, many clusters merged as they became larger. The merged clusters in time-interval 2, as well as clusters identified only on this time interval, were represented by letters (**Figure 2C**). At total, almost 90% of lead-treated ES-NP cell interactome was altered in time-interval 2. All upregulated clusters in time-interval 1 expanded in time-interval 2: clusters 1, 2, and 3 merged into cluster A and cluster 4 drastically increased in time-interval 2. Additionally, upregulated clusters increased their transcriptional differences against the control group (black line in **Figure 2C**). Downregulated peaks decreased against the control group when compared to time-interval 1. It indicates a strong early modulation of downregulated clusters that decreases with time-interval transition. Cluster average connectivity is more equalized in time-interval 2 where the upregulated clusters enhanced their average connectivity (**Figure 2D**).

**Figure 3:**
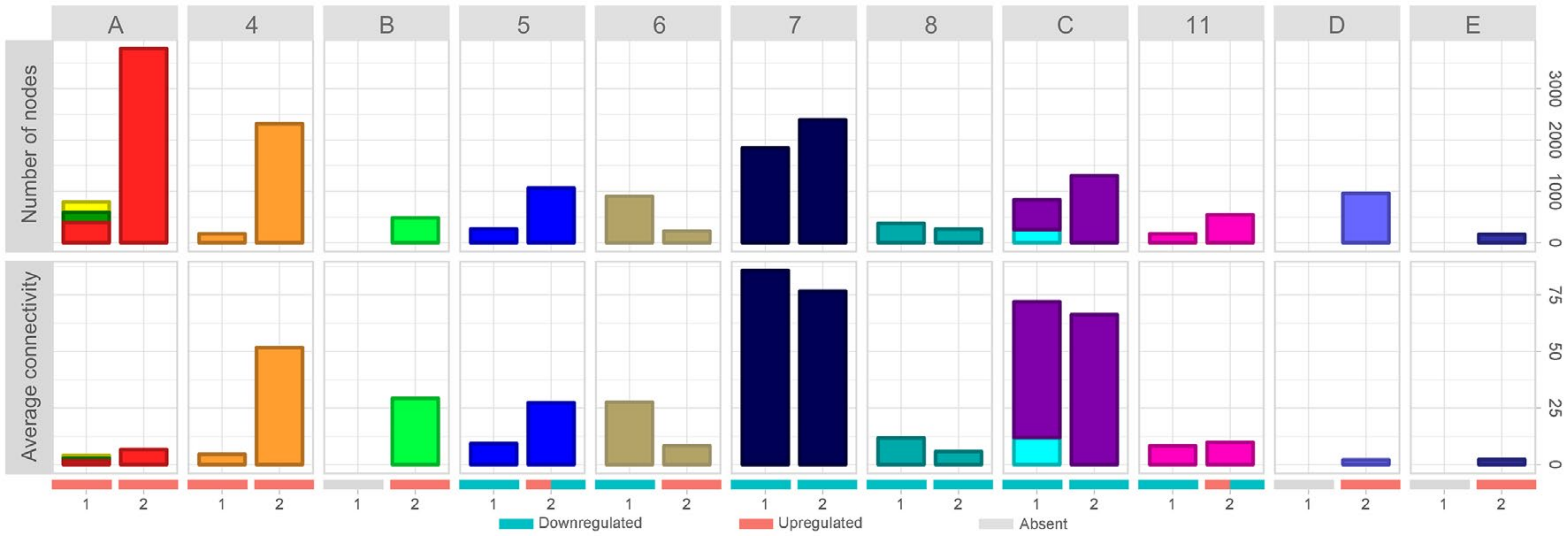
Evolution of network average connectivity and number of nodes. Cluster labels refer to time-interval 2. Clusters labeled with numbers were identified in time-intervals 1 and 2; merged clusters, as well as new clusters, were labeled with letters. Bar colors refer to cluster colors at time-intervals 1 and 2, respectively. Stacked bar charts represent clusters of time-interval 1 merged on time-interval 2. Numbers at the X-axis identify the time interval. Red, blue, and gray bars denote the expression status (upregulated clusters, downregulated clusters, and absent cluster at that time interval, respectively). Clusters 5 and 11 have both upregulated and downregulated expression patterns on time-interval 2. Top panels show the number of nodes variation through time intervals. Bottom panels represent the average connectivity (< k>) variation through time intervals.

The dynamic involving the number of nodes and the average connectivity of each cluster in the different time intervals can be better observed in **Figure 3**. It is possible to observe that all upregulated clusters in time-interval 1 strongly increased in number of nodes in time-interval 2 (**Figure 3**, clusters A and **4**). While cluster 4 highly increased the average connectivity, clusters 1, 2, and 3 (merged as cluster A in time-interval 2) average connectivity barely changed. Clusters identified as downregulated in time-interval 1 also increased in time-interval 2, except by clusters 6 and 8. However, the expansion observed in downregulated clusters was smaller when compared with the expansion experienced by upregulated clusters in time-interval transition. In spite of increasing number of nodes, clusters identified as downregulated in time-interval 1 showed a slight decrease in average connectivity in time-interval 2, except by clusters 5 and 11 that have a particular dynamic on time-interval transition: they started as downregulated clusters on time-interval 1 and expanded on time-interval 2, and then presented down- and upregulated portions on the transcriptogram. The expansion in the number of nodes observed in clusters 5 and 11 was followed by an increase in average connectivity.

**Figure 4:**
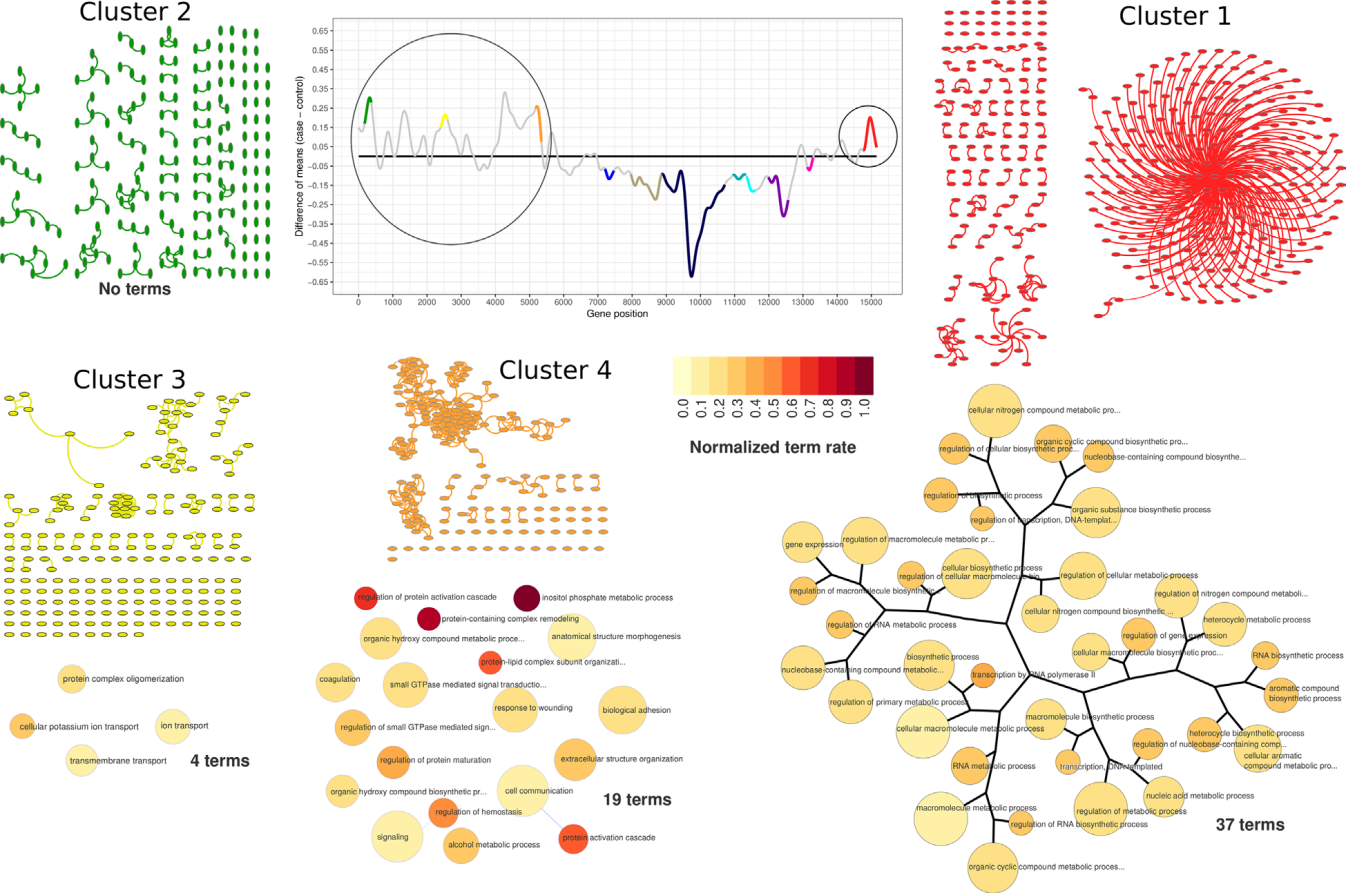
PPI network visualization and term dendrograms of upregulated clusters. Transcriptogram, cluster names, and cluster colors correspond to time-interval 1. The circle over the transcriptogram indicates the highlighted upregulated clusters. PPI network nodes are represented as ellipsis. Upregulated cluster networks are small and poorly connected, as it is possible to observe in network representation. Dendrograms refer to clusters enriched GO hierarchy. Dendrogram node sizes are proportional to the number of terms held by each GO term. Dendrogram colors represent the normalized terms occupation rate, where darker colors indicate the ratio between the number of cluster genes overlapping to the term genes and the number of term genes, normalized by the maximum ratio found in the cluster.

### Functional Enrichment of Upregulated Clusters in Time-Interval 1

According to our data, lead treatment was able to impair ES-NP cell global expression in both time intervals, either by increasing or decreasing gene group transcription. As shown in **Figure 3**, clusters identified as upregulated in time-interval 1 have low average connectivity. **Figure 4** shows the PPI network of each cluster identified as upregulated in time-interval 1 and, except by a few nodes in cluster 1 and cluster 4, the nodes of upregulated clusters are poorly connected. We then performed Gene Ontology enrichment analysis of each upregulated cluster. *Transcriptogramer* R/Bioconductor package implements a Gene Ontology enrichment analysis. For each differentially expressed cluster, we calculated the distance, all-against-all, of significantly enriched GO terms. The distance is based on the Jaccard Index between any GO term pair. The resulting adjacency matrix is then used to plot a hierarchical network of significantly enriched GO terms associated with each cluster identified as differentially expressed in transcriptogram analysis (see the *Materials and Methods* section for details). GO enrichment analysis of cluster 1 resulted in 37 significantly enriched GO terms. The terms were associated with molecule biosynthesis, especially RNA molecules, transcription, and gene expression regulation (**Figure 4** and **Supplementary Table S1**). The four cluster 3 enriched terms were related to transmembrane transport, protein complex oligomerization, ion transport, and cellular potassium ion transport. Cluster 4 had 19 enriched terms, which were related with cellular adhesion and signaling transduction, especially GTP-mediated cascades. There was no GO term significantly enriched associated with cluster 2. It is important to note that only cluster 1 GO terms were sufficiently associated with each other to form a hierarchical network. Accordingly, lead-induced increase in gene expression in time-interval 1 seems not to be related to massive biochemical system modulation.

### Functional Enrichment of Downregulated Clusters in Time-Interval 1

**Figure 5:**
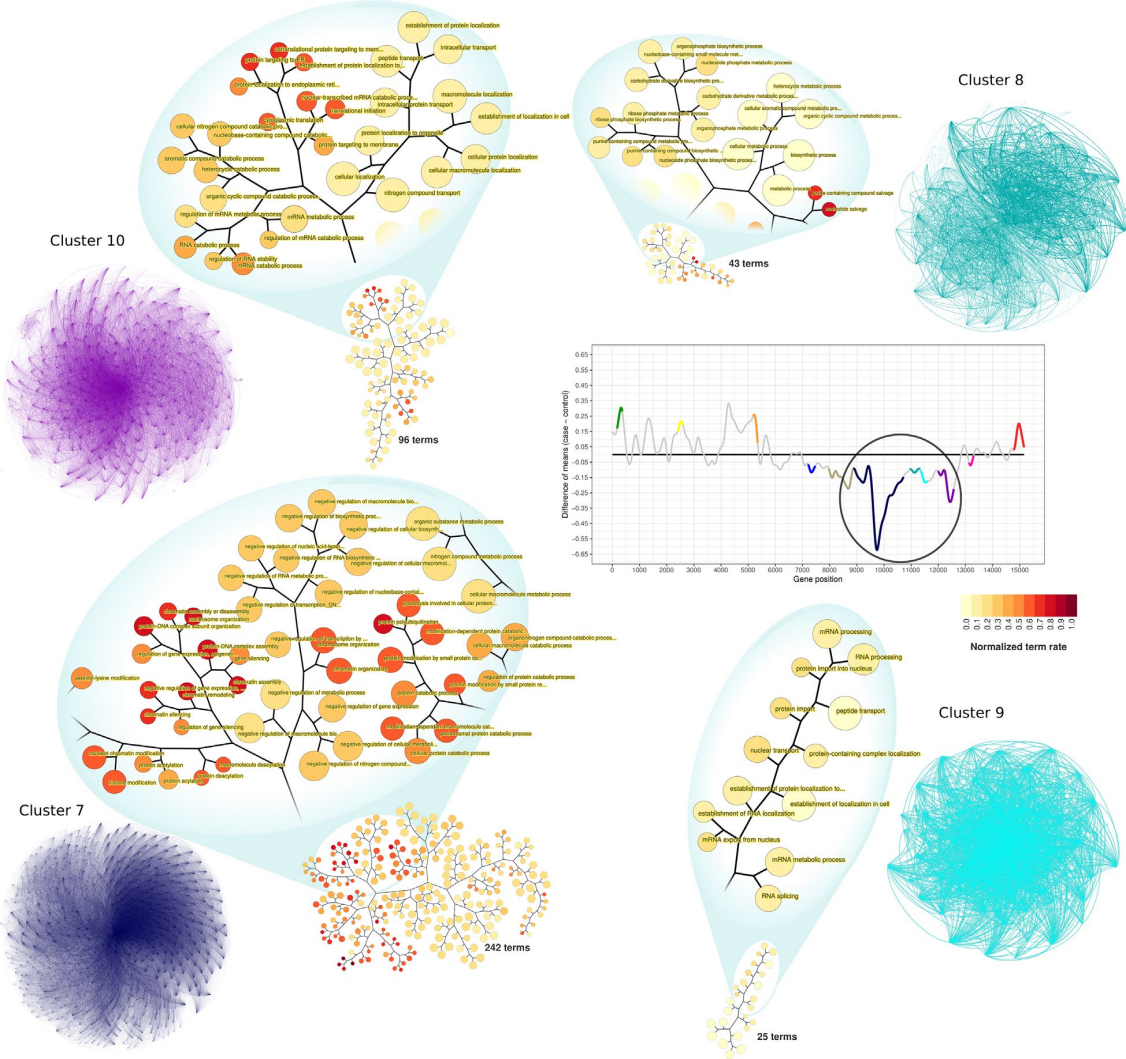
PPI network visualization and terms dendrograms of downregulated clusters. Transcriptogram, clusters names, and cluster colors correspond to the time-interval 1. The circle over the transcriptogram indicates the highlighted downregulated clusters. Nodes are the cluster’s relevant proteins but are not explicitly represented. The confluence of edges identifies them. Dendrograms refer to the clusters enriched GO hierarchy. Node sizes are proportional to the numbers of terms held by each GO term. Dendrogram colors represent the normalized term occupation rate, where darker colors indicate the ratio between the number of cluster genes overlapping to the term genes and the number of term genes, normalized by the maximum ratio found in the cluster. Zoom areas on the dendrogram show relevant terms based on the occupation rate index.

Among the clusters identified as downregulated on time-interval 1, clusters 7 to 10 represent large and highly connected networks (**Figure 5**). Terms significantly enriched in those clusters include processes related to RNA metabolism and gene expression regulation. GO enrichment analysis of cluster 7 resulted in 242 enriched GO terms. Among them, there were terms related to chromatin organization, DNA-damage cellular response, negative regulation of cell cycle, negative regulation of transcription processes, and negative regulation of gene expression. GO terms associated with cluster 8 (43 terms) were related to RNA biosynthesis, particularly of non-coding RNAs. Cluster 9 terms (25 at total) were associated with mRNA processing and protein biosynthesis and transport. Similarly, cluster 10 was enriched with terms related to protein biosynthesis, also having terms of RNA catabolism regulation (96 terms, at total).

Clusters 5, 6, and 11 are characterized by the shifting expression between time intervals (**Figure 2**). PPI networks and enriched terms are presented in Supplementary Material Online (**Supplementary Figure S6**). GO enrichment analysis of cluster 5 resulted in 29 enriched GO terms associated with vesicle formation, transport, and exocytosis. GO enrichment analysis of cluster 6 resulted in 188 terms mainly related to cell cycle regulation and cytoskeleton organization. Finally, cluster 11 enrichment results in 20 terms related to oxidative metabolism and ATP metabolic process. In contrast with upregulated clusters, downregulated cluster GO terms are numerous (**Supplementary Table S1**). Additionally, enriched terms in each downregulated cluster are functionally related to each other inside the cluster, as can be observed by the GO term hierarchical networks (**Figure 5** and **Supplementary Figures S8**–**S17**).

### Functional Enrichment in Time-Interval 2

The massive alteration in transcription profile observed on time-interval 2 reflects on cluster network properties, especially the number of nodes and connectivity, and also in their enriched GO terms. Time-interval 2 total interactome enlargement is followed by an increase in the total number of enriched GO terms (**Supplementary Figure S7**). In the first time interval, the majority of cluster terms are related to RNA biosynthesis and metabolism, pointing to an acute disturbance of transcriptional pattern induced by lead exposure. In the second time interval, it is difficult to identify a unique biological process affected, pointing to a massive alteration in cell metabolism.

## Discussion

Lead poisoning effects are broad and affect several cellular systems (Neal and Guilarte, 2010; Mitra et al., 2017). Among them, neurological effects of lead poisoning are critical, especially during development (Lidsky and Schneider, 2003; Martino and Pluchino, 2006; White et al., 2007; Sanders et al., 2009). According to our results, lead treatment was able to strongly decrease the expression of several cellular systems in the first 11 days of treatment, resulting in a massive transcriptional impairment observed at the end of the treatment. Lead is classified as a potent neurotoxin, interfering with specific brain areas and neuronal pathways, being particularly harmful to children. Early and chronic exposure, even to low lead levels, were associated with brain damage, abnormal neurodevelopment, impairment of IQ levels, neuropsychological dysfunction, as well as behavioral disorders (Meyer et al., 2008; Schneider et al., 2012; Schnur and John, 2014). The results observed here suggest that lead neurotoxicity during development could be associated with a huge transcriptional impairment in developing nerve cells.

Cell differentiation requires strict regulation of gene expression in order to ensure correct cell fate. Some studies report lead exposure impact on embryonic stem cells differentiation (Huang and Schneider, 2004; Abdullah et al., 2014; Senut et al., 2014). In a study involving embryonic stem cells cultivated with lead acetate 10 μM for 5 days, the authors observed significant proliferation inhibition in neurospheres collected from rat brain regions (Huang and Schneider, 2004). In contrast, Senut et al. (2014) observed exposure to lead acetate (0.4 and 1.9 μM) did not affect the differentiation of human embryonic stem cells (hESCs) to NPCs. However, these authors showed that lead could change neuronal features such as neurites and branching when NPCs differentiate to neurons, while also altering the methylation pattern of genes involved in neurogenetic signaling pathways (Senut et al., 2014). The study conducted by Jiang and collaborators has investigated the effect of lead treatment in ES-NP cells during differentiation to neuronal cells to evaluate lead-caused neurotoxicity (Jiang et al., 2017). However, there is no assay in the original paper to assess the differentiation process efficiency. We measured the expression of classical NPCs and neuronal marker genes during the experiment to evaluate the differentiation of ES-NP cells in neuronal cells. Overall, the NPC markers decrease their expression and the neuronal markers increased their expression throughout the experiment in both control and treated cells. This pattern is consistent with a successful differentiation process. However, the marker expression dynamics were not the same among treated and control (as shown in **Supplementary Figures S4** and **S5**), which suggests that the differentiation process was impaired in some extent.

Jiang and collaborators investigated the expression of specific genes to identify possible lead-associated disease development biomarkers. They found transcriptional alterations in genes involved with cell–cell signaling, cell development, and cell cycle, as well as response to stress and DNA damage. The authors have described early modulation of cell cycle genes, neurotransmitter transport, and organelle fission. Later modulated genes were associated to positive regulation of biological, metabolic, and immune system processes (Jiang et al., 2017). Here, we investigate the same data from a systemic perspective, since *transcriptogramer* provides a global view of cellular metabolism by indicating functionally associated gene sets with altered expression in a given biological condition. The approach used here allowed to globally assess the transcriptional impact of lead treatment in impairing entire biochemical systems, and therefore, our results are complementary to the results of Jiang and collaborators.

According to our results, lead treatment was able to decrease entire cellular systems in time-interval 1 as denoted by the large and densely connected downregulated networks, representing biological functions related to cell cycle regulation, chromatin modification, cytoskeleton organization, RNA biosynthesis and transcription regulation, protein biosynthesis and protein transport, vesicle formation, and exocytosis. When taking together, the downregulation of those biochemical systems suggests deregulation in the vital cellular process, which might result in a differentiation impairment. Several GO terms in cluster 8 are related to nucleotide metabolic process and nucleotide salvage, especially purine-containing ribose-phosphate nucleotides, such as ATP and GTP. It might reflect, to some extent, into impairment in energetic metabolism. This illustrates lead interference in the various layers of gene expression regulation, from chromatin modifications to cellular exocytosis. On the other hand, upregulated networks in time-interval 1 were composed of few nodes poorly connected. It could indicate an initial activation of systems, which will be massively modulated later, as observed in time-interval 2. Reinforcing that, several GO terms significantly enriched in time-interval 1 upregulated clusters were associated with transcriptional modulation, such as ion transport, GTP-mediated cascades, and gene expression regulation itself. On the second time interval, upregulated cluster networks were bigger and more connected when compared to time-interval 1, suggesting a late modulation of cellular processes mediated by upregulated clusters.

Several biochemical systems that were downregulated in time-interval 1 (e.g., cycle regulation, transcription regulation, protein biosynthesis, and nucleotide-phosphate metabolism) are still downregulated in time-interval 2. Considering time-interval transition, both transcriptograms show an extensive time-dependent alteration of ES-NP cell transcription pattern. Additionally, the behavior of neuronal marker expression in control and treated samples reinforces those observations. Four of the six neuronal markers (i.e., *GAD1*, *GAD2*, *NEUROD6*, and *TH*) were not significantly altered in time-interval 1, while all the evaluated neuronal markers were significantly altered in time-interval 2 (always comparing treated samples with control samples). Interestingly, some of them are upregulated in treated samples (*RBFOX3* and *TH*), while others are downregulated in treated samples (*GAD1*, *GAD2*, and *NEUROD6*). All the NPC markers evaluated are downregulated in treated samples in time-interval 1, agreeing with the huge downregulation of several biochemical systems. One of the downregulated systems in time-interval 1 is the *mTOR* signaling proteins (GO:0031929, **Supplementary Table S1**) found in cluster 6. *mTOR* signaling cascade is related to neuronal and glial development, learning, memory, and synaptic plasticity, being activated by the brain-derived neurotrophic factor (*BDNF*) (Lipton and Sahin, 2014; Switon et al., 2017). In a previous study, Neal et al. (2010) demonstrated that lead exposure decreased *BDNF* levels in hippocampal neurons. The authors also observed that exogenous treatment with *BDNF* applied to lead-exposed cells recovered presynaptic protein levels and vesicular neurotransmitter release, which suggests a presynaptic mechanism for lead poisoning (Neal et al., 2010; Stansfield et al., 2012). Synaptic plasticity is affected by lead during neurodevelopment and *mTOR* signaling cascade could be a biological target to lead poisoning (White et al., 2007). However, the interaction of *mTOR* and *BDNF* signaling pathways in lead poisoning should be properly tested in future studies, which might help to clarify lead neurotoxicity mechanism.

The diverse and systemic effects of lead poisoning reflect lead ability to impair several cellular components, such as receptors, membranes, and transcription factors, disturbing cell function (de Souza et al., 2018). Lead interference in gene expression is reported in several studies, specially suggesting gene expression disturbance as the underlying mechanism for lead neurotoxicity (Nihei and Guilarte, 1999; Bouton et al., 2001; Nihei and Guilarte, 2001; Kasten-Jolly et al., 2011; Kasten-Jolly et al., 2012; Schneider et al., 2012). The results showed here demonstrated a lead-induced transcriptional impairment during ES-NP cell differentiation, affecting several biochemical systems crucial to neurodevelopment, such as *mTOR* signaling. The comprising in transcriptional homeostasis is compatible with neuronal and progenitor marker expression dynamics. Therefore, it is reasonable to assume that the normal development of neuronal cells could be impaired in lead intoxication, especially by altering transcription homeostasis. However, it is difficult to determine the real consequence of lead in central nervous system development *in vivo*, and further studies are needed to totally elucidate lead poisoning neuronal impact.

## Data Availability

Data supporting this paper can be accessed through https://github.com/LabBiosystemUFRN/LeadNPC

Datasets analyzed for this study can be found in the NCBI-Bioproject Accession PRJNA330909 (GEO: GSE84712) at https://www.ncbi.nlm.nih.gov/geo/query/acc.cgi?acc=GSE84712. 

## Author Contributions

CR and IS performed the analysis and wrote the manuscript. DM collected the data and performed the analysis. RO and DI performed the analysis and organized the supplementary material. RD and RA designed the study and wrote the manuscript. All authors reviewed and approved the final manuscript. CR and IS have contributed equally to this work.

## Funding

This work has been financed by the governmental Brazilian agency National Council for Scientific and Technological Development (CNPq, Portuguese: Conselho Nacional de Desenvolvimento Científico e Tecnológico), grant 444856/2014-5. The scholarships were financed by governmental Brazilian agency Coordination for the Improvement of Higher Education Personnel (CAPES–Portuguese: Coordenação de Aperfeiçoamento de Pessoal de Nível Superior).

## Conflict of Interest Statement

The authors declare that the research was conducted in the absence of any commercial or financial relationships that could be construed as a potential conflict of interest.
