# Supplementary material for: Systems Biology-Based Analysis Indicates Global Transcriptional Impairment in Lead-Treated Human Neural Progenitor Cells
Source: Front Genet. 2019 Sep 10;10:791. doi: 10.3389/fgene.2019.00791 (PMC6748217; doi:10.3389/fgene.2019.00791)
Supplement: Supplementary file 1 [file DataSheet_1.pdf]

Supplementary Material

SUPPLEMENTARY FIGURES

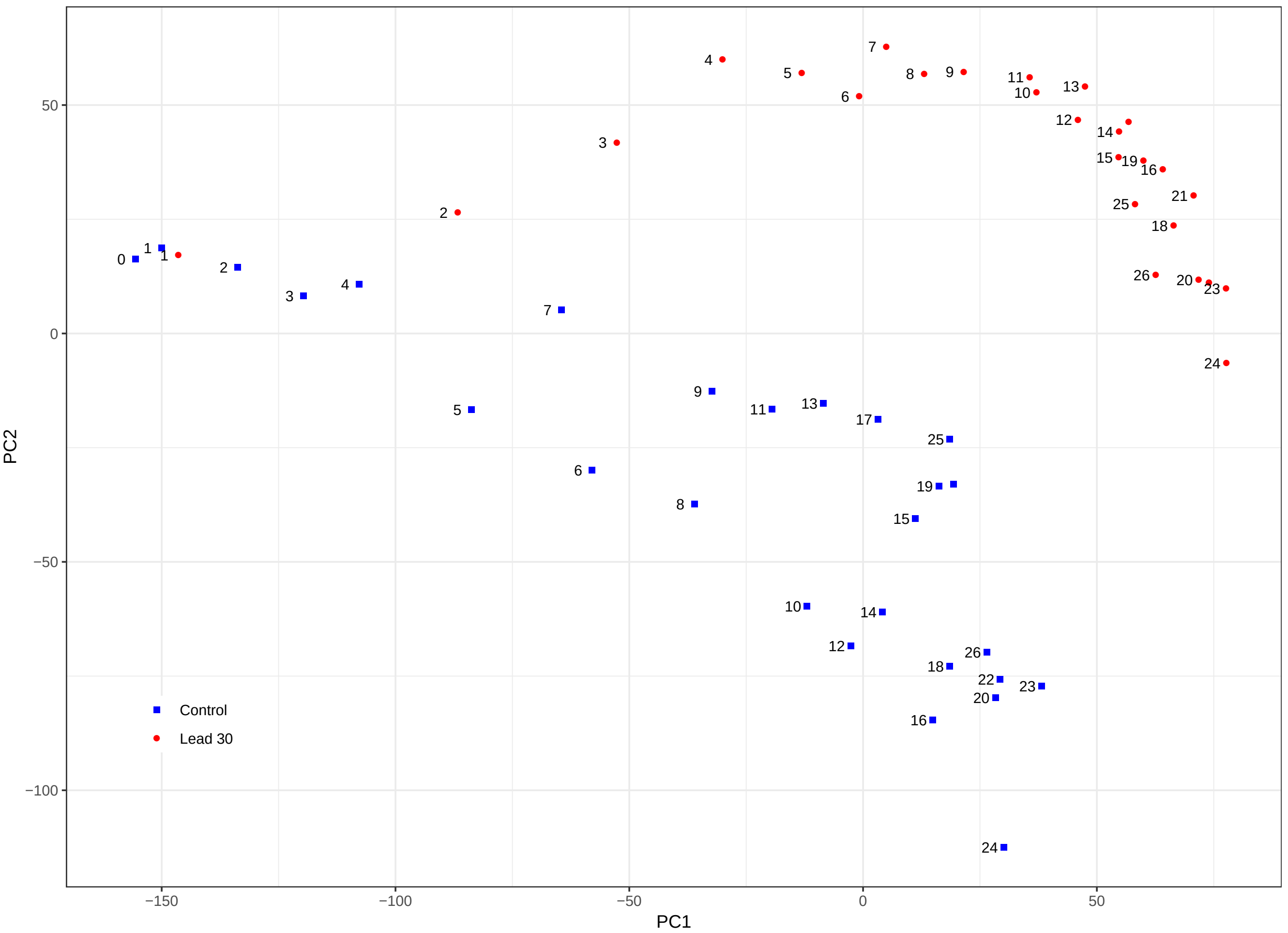

Figure S1: PCA after expression normalization. PC1xPC2. Blue dots designate the control, and red dots the 30μM samples. The number indicates the time of cell exposition to lead.

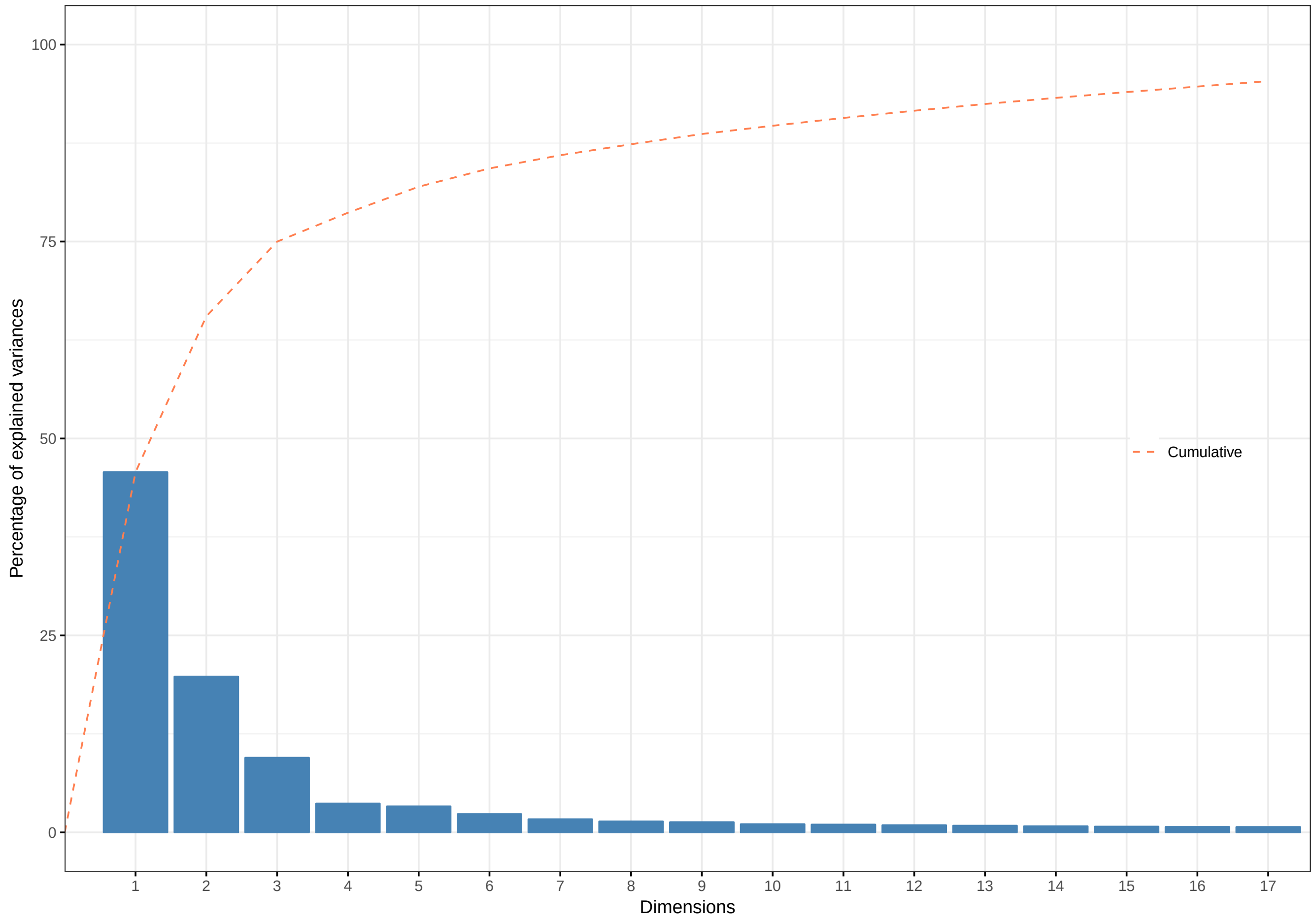

Figure S2: Percentage of explained variances of Lead30 samples used for clusterization. Blue bars indicate the explained variances of the first 17 components and the orange line it cumulative total (95.372%).

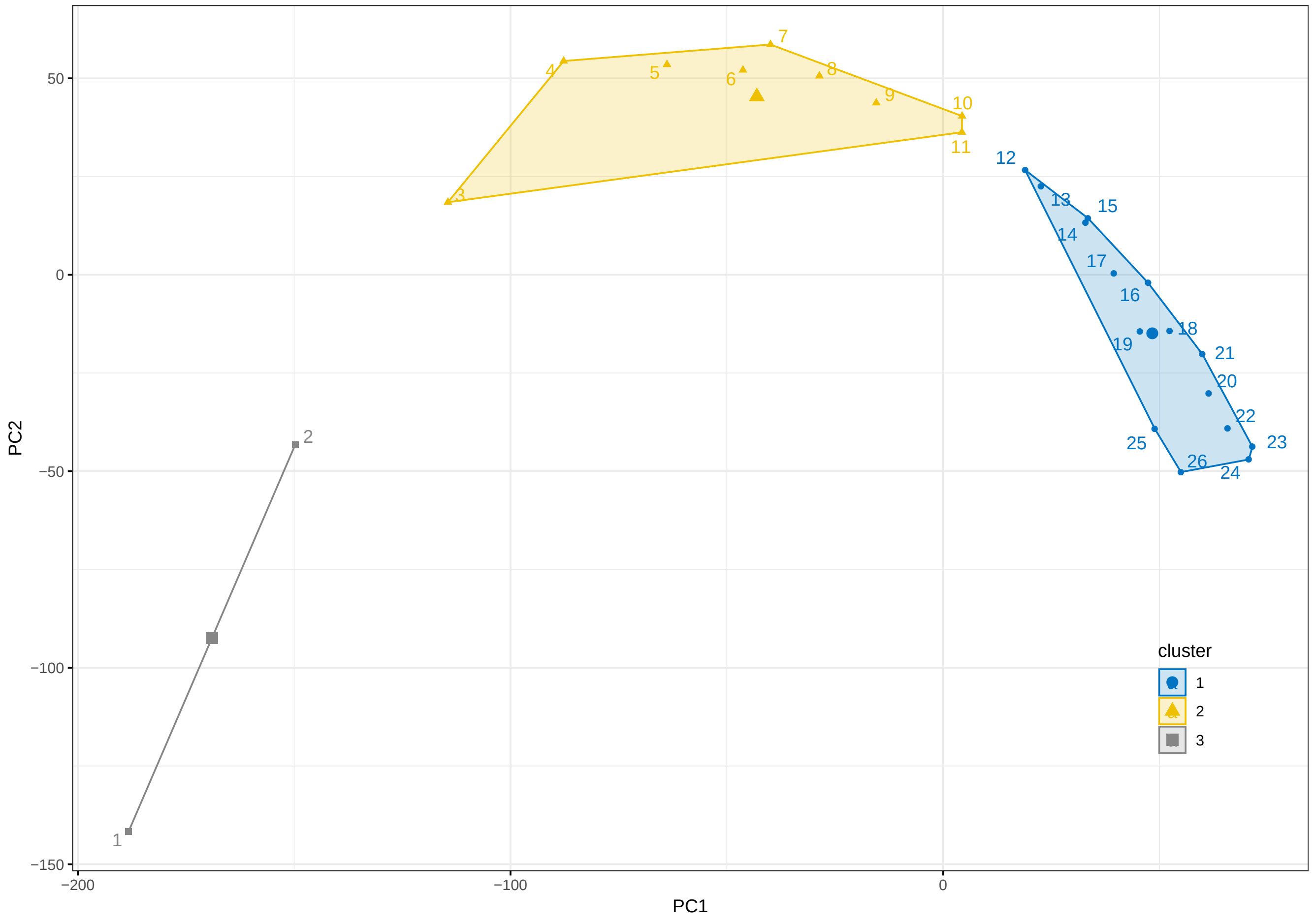

Figure S3: PCA clusterization of the Lead30 samples. Colors designate the different clusters found using the R package FactoMineR's HCPC function whit autodetection of the number of clusters (k).

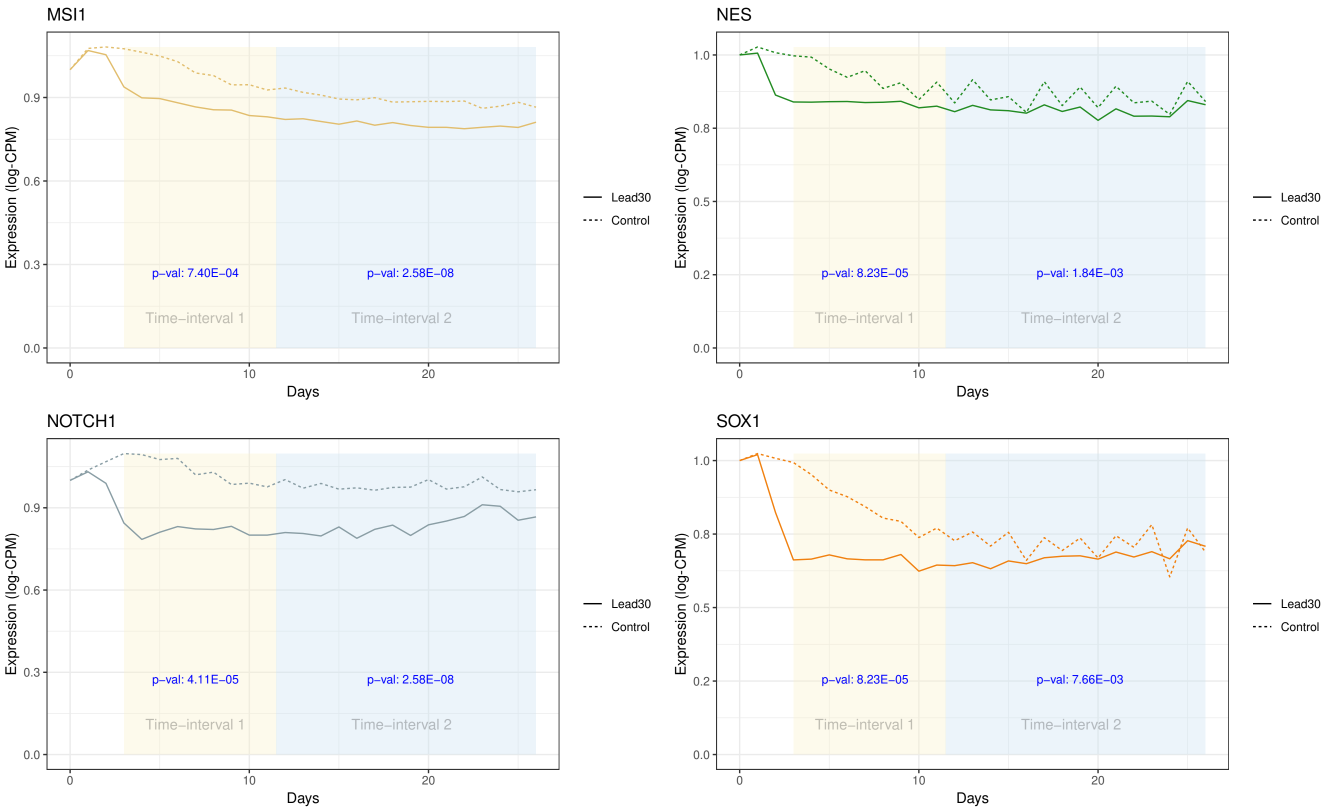

Figure S4: NPC cells markers progression time-line. Graphics show the time points of control group expression values for NPC cells markers (dotted lines), and lead-treated samples expression values (solid lines). Colors represent distinct markers. Numbers at X-axis identify the days of treatment. Numbers at Y-axis represent the expression values of samples, in log-CPM. Shaded areas inside graphical area delimit the time-intervals 1 and 2, from day 3 to 11, and day 12 to 26, respectively. Kolmogorov-Smirnov test comparing the entire timeline of lead-treated and control samples determine significant differences between datasets with p-values lower than 6×10^{-5} for all markers. Kolmogorov-Smirnov test p-values of comparison between lead-treated and control samples in time-interval 1 and time-interval 2, when taken in isolation, have it p-values corrected by FDR annotated inside the graphic. Blue p-values indicate significant differences between distributions (p-value ≤ 0.01).

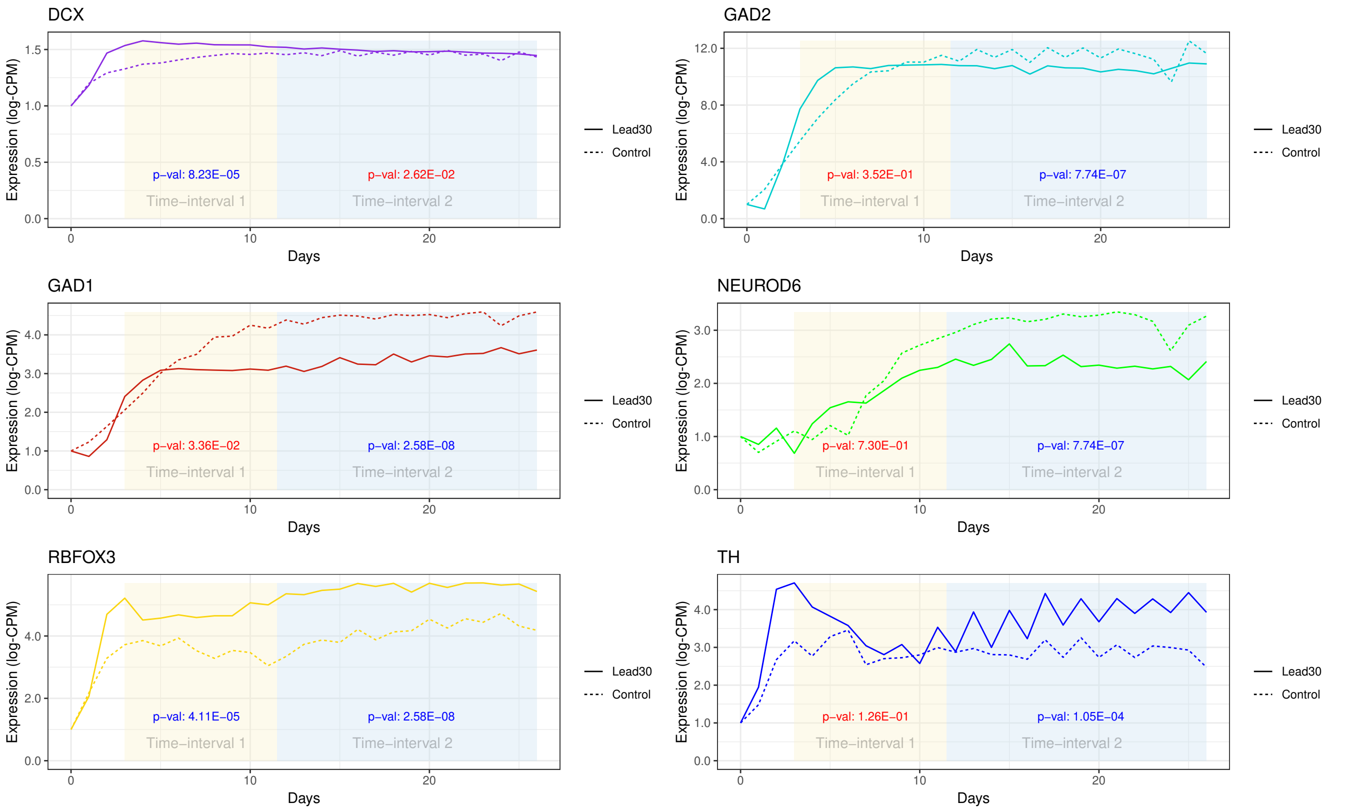

Figure S5: Neuronal cells markers progression time-line. Graphics show the time points of control group expression values for Neuronal cells markers (dotted lines), and lead-treated samples expression values (solid lines). Colors represent distinct markers. Numbers at X-axis identify the days of treatment. Numbers at Y-axis represent the expression values of samples, in log-CPM. Shaded areas inside graphical area delimit the time-intervals 1 and 2, from day 3 to 11, and day 12 to 26, respectively. Kolmogorov-Smirnov test comparing the entire timeline of lead-treated and control samples determine significant differences between datasets with p-values lower than 6×10^{-5} for all markers. Kolmogorov-Smirnov test p-values of comparison between lead-treated and control samples in time-interval 1 and time-interval 2, when taken in isolation, have it p-values corrected by FDR annotated inside the graphic. Red p-values indicate non-significant differences between distributions and blue p-values indicate significant ones (p-value ≤ 0.01).

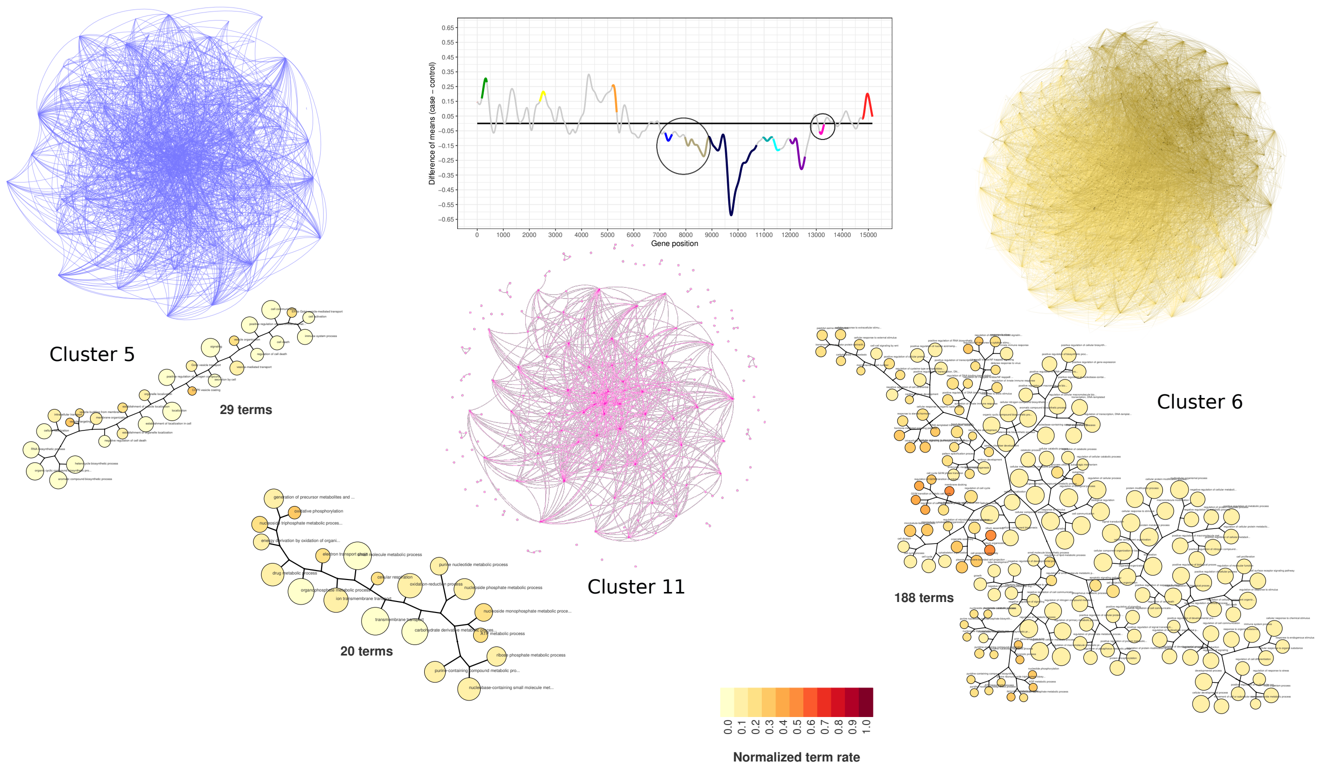

Figure S6: Connectivity and GO terms of clusters 5, 6, and 11. Transcriptogram, cluster's names, and colors correspond to the time-interval 1. Top graph of each cluster represents the connectivity of PPI networks. Nodes are the cluster's relevant proteins but are not explicitly represented. The confluence of edges identifies them. Botton graph of each cluster is a dendrogram and refers to the cluster's enriched GOs hierarchy. Circles represent each enriched GO term. Node sizes are proportional to the numbers of terms held by each GO term. Dendrogram colors represent the normalized terms occupation rate, where dark colors indicate a larger number of genes detected over all genes belonging to the GO. Distance between two nodes is proportional to the number of genes they have in common.

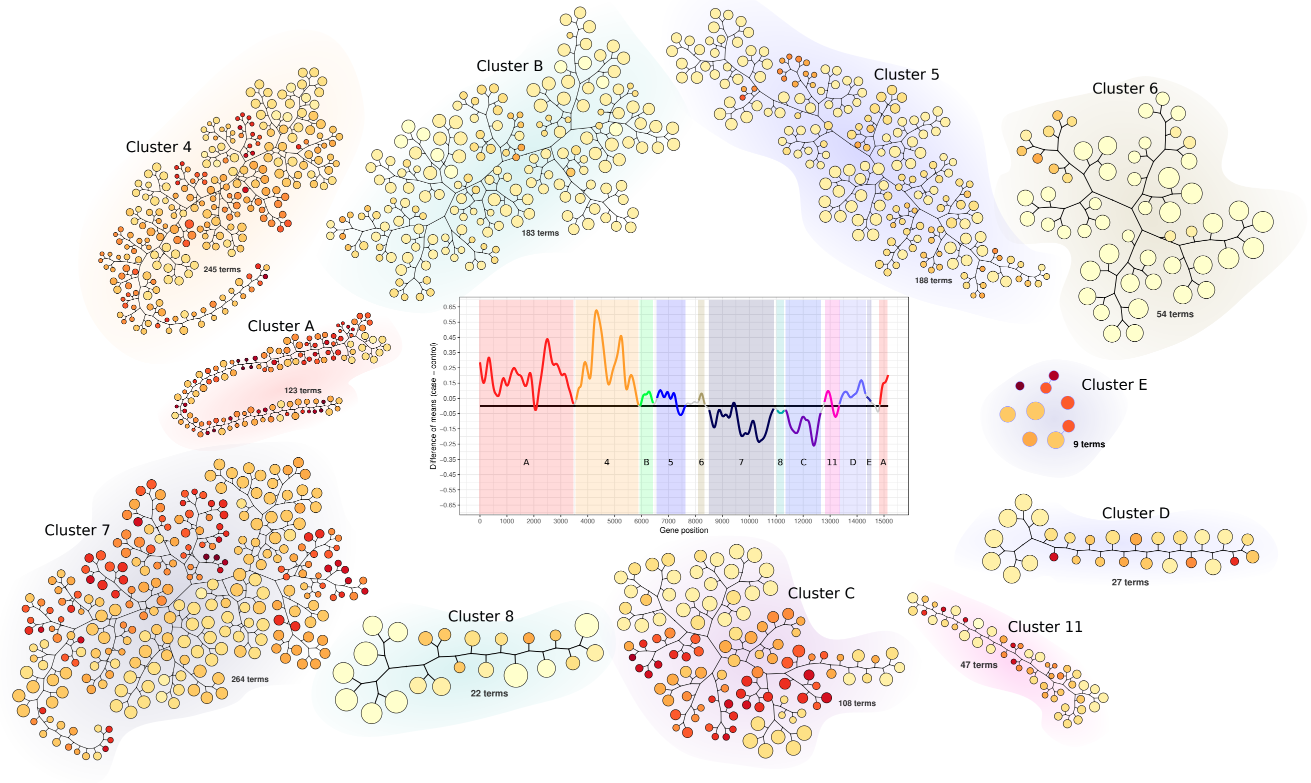

Figure S7: Connectivity and GO terms of time-interval 2. Transcriptogram, cluster's names, and colors correspond to the time-interval 2. Dendrograms refers to the cluster's enriched GOs hierarchy. Its node sizes are proportional to the numbers of terms held by each GO term. Dendrogram colors represent the normalized terms occupation rate, where dark colors indicate a larger number of genes detected over all genes belonging to the GO.

Figures S6 to S15 are dendrograms of clusters 1 to 11 of time-interval 1, respectively, and refers to the cluster's enriched GOs hierarchy. Circles represent each enriched GO term and are labeled by it respective description. Node sizes are proportional to the numbers of terms held by each GO term. Dendrogram colors represent the normalized terms occupation rate, where dark colors indicate a larger number of genes detected over all genes belonging to the GO. Distance between two nodes is proportional to the number of genes they have in common. Cluster 2 do not have a dendrogram because it had no GO terms enriched.

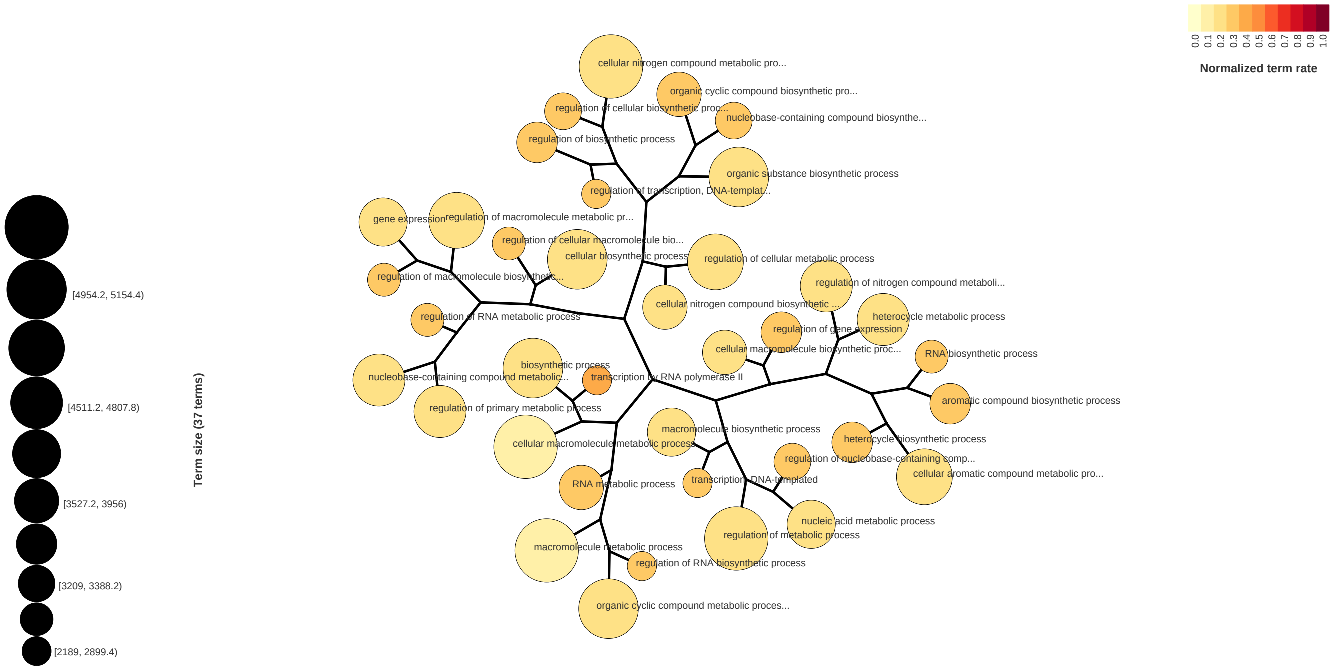

Figure S8: Complete dendrogram of cluster 1 – time-interval 1

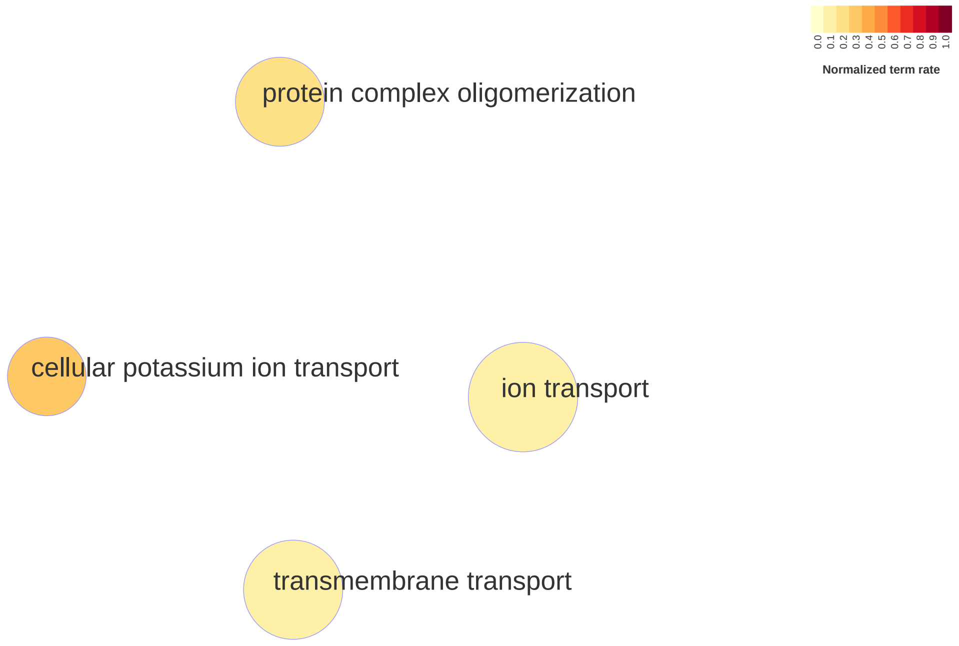

Figure S9: Complete dendrogram of cluster 3 – time-interval 1

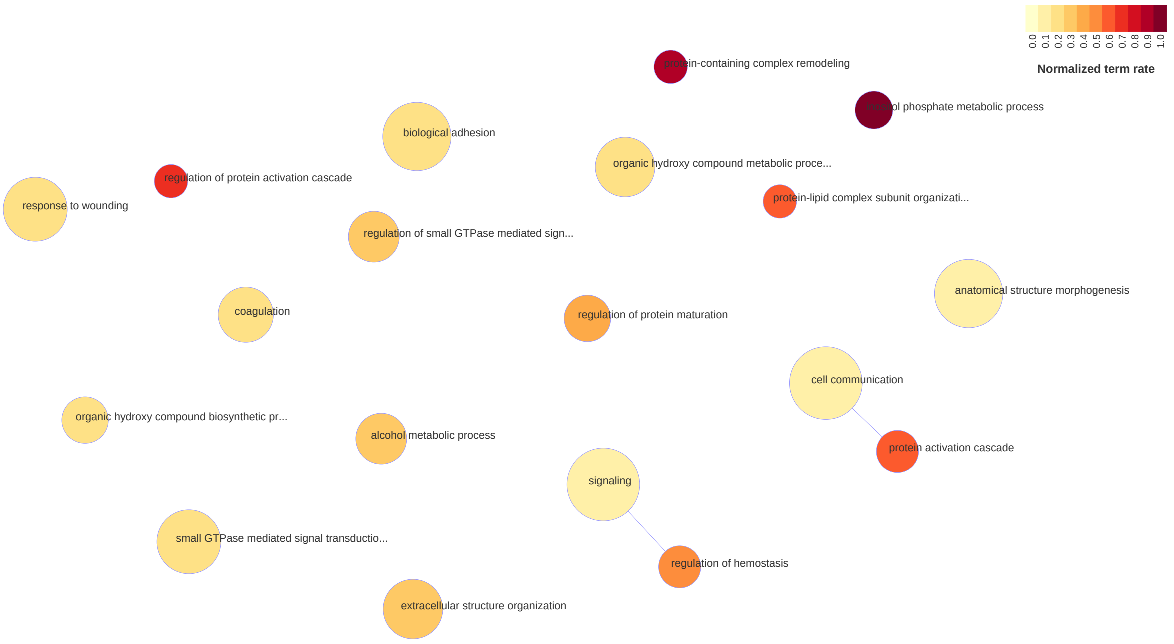

Figure S10: Complete dendrogram of cluster 4 – time-interval 1

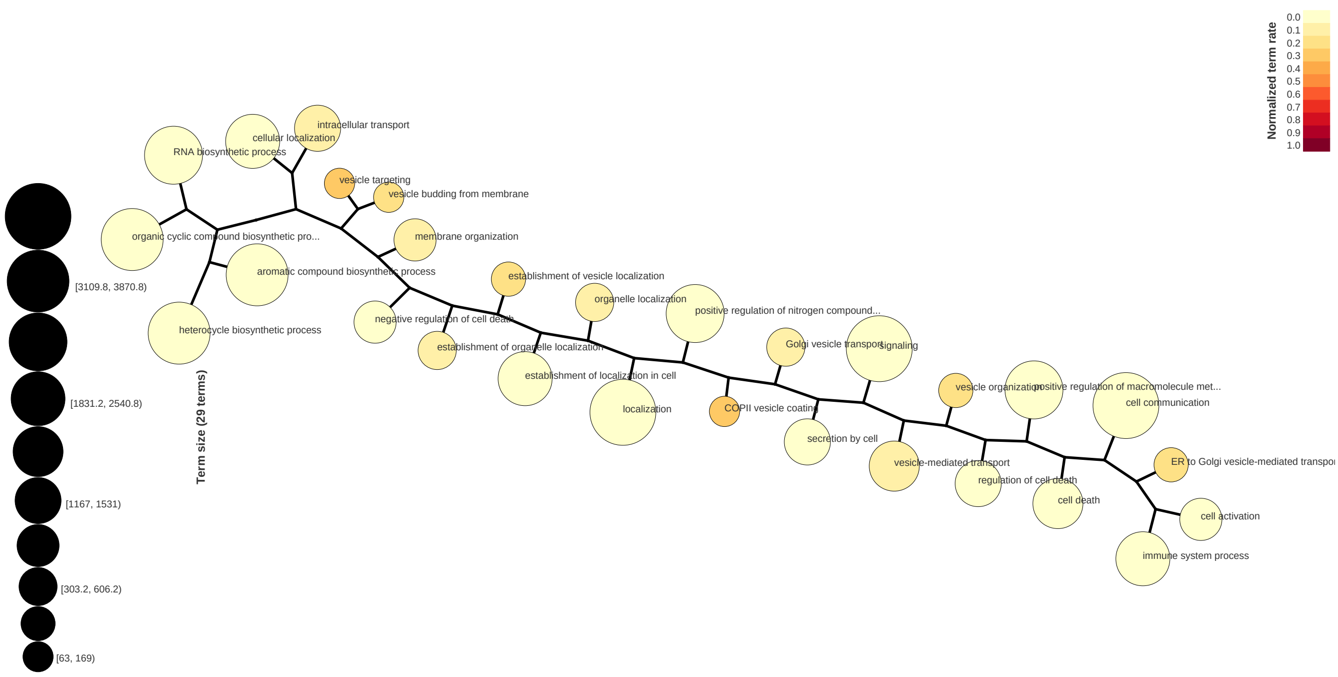

Figure S11: Complete dendrogram of cluster 5 – time-interval 1

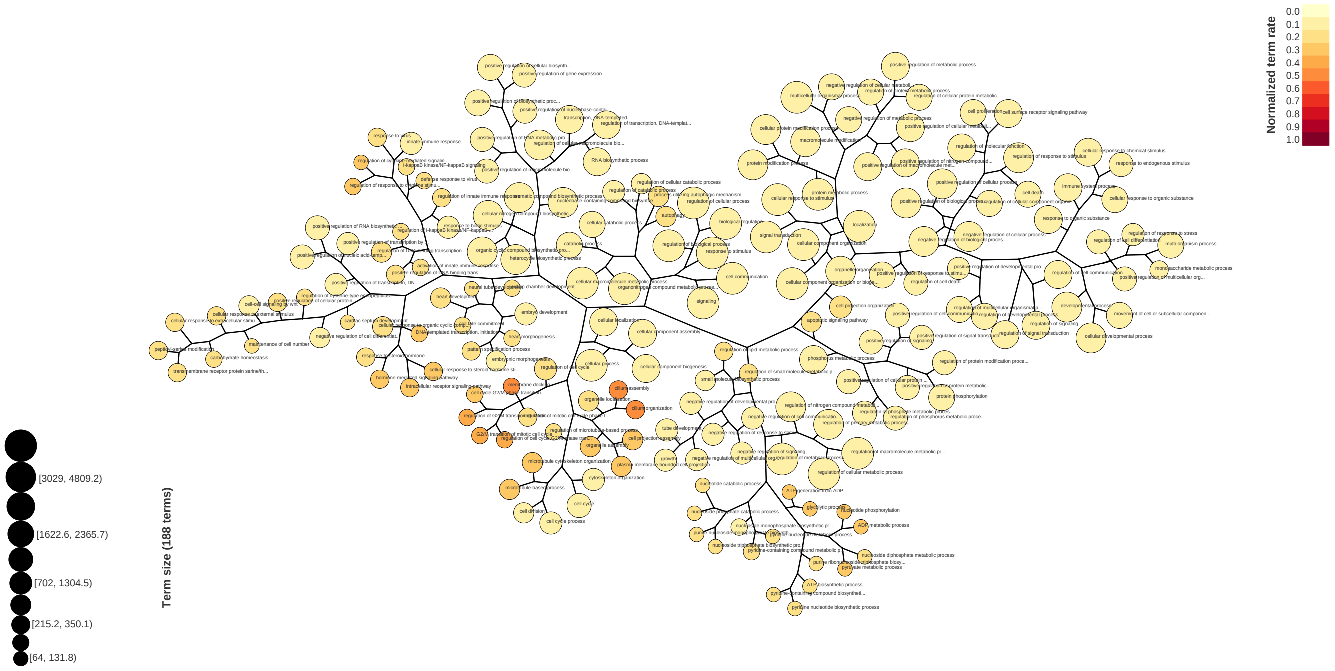

Figure S12: Complete dendrogram of cluster 6 – time-interval 1

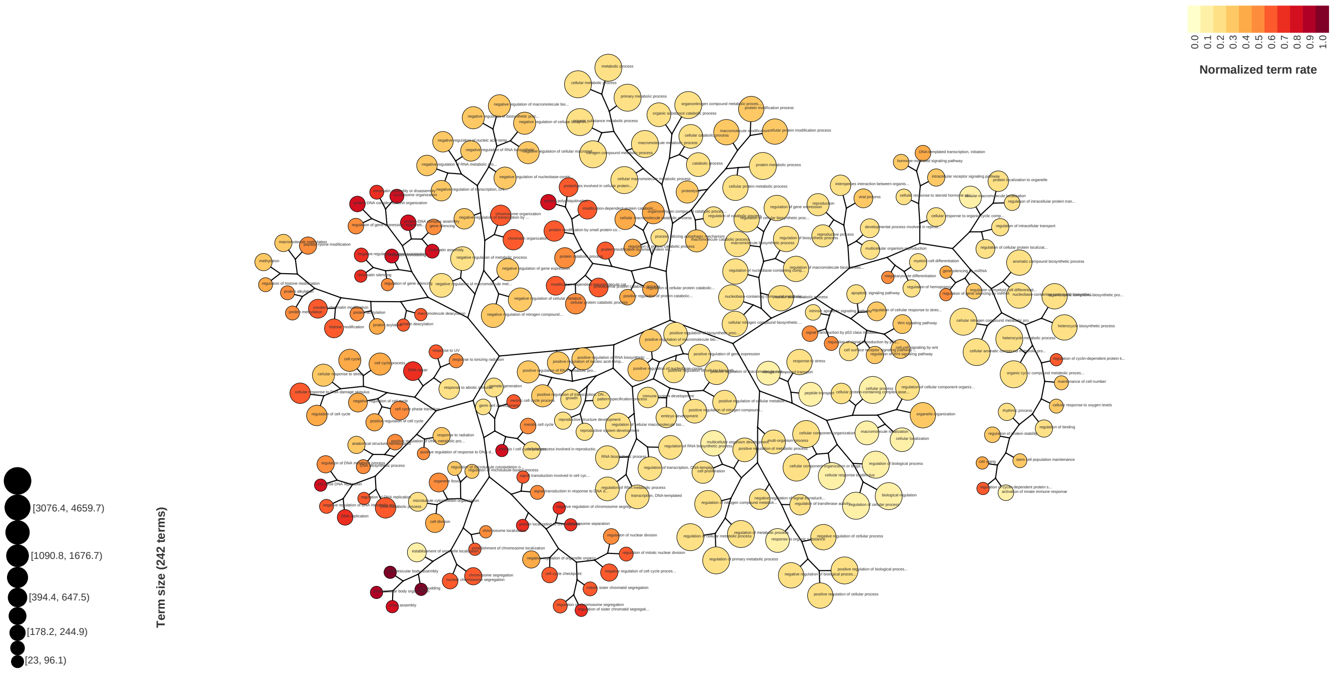

Figure S13: Complete dendrogram of cluster 7 – time-interval 1

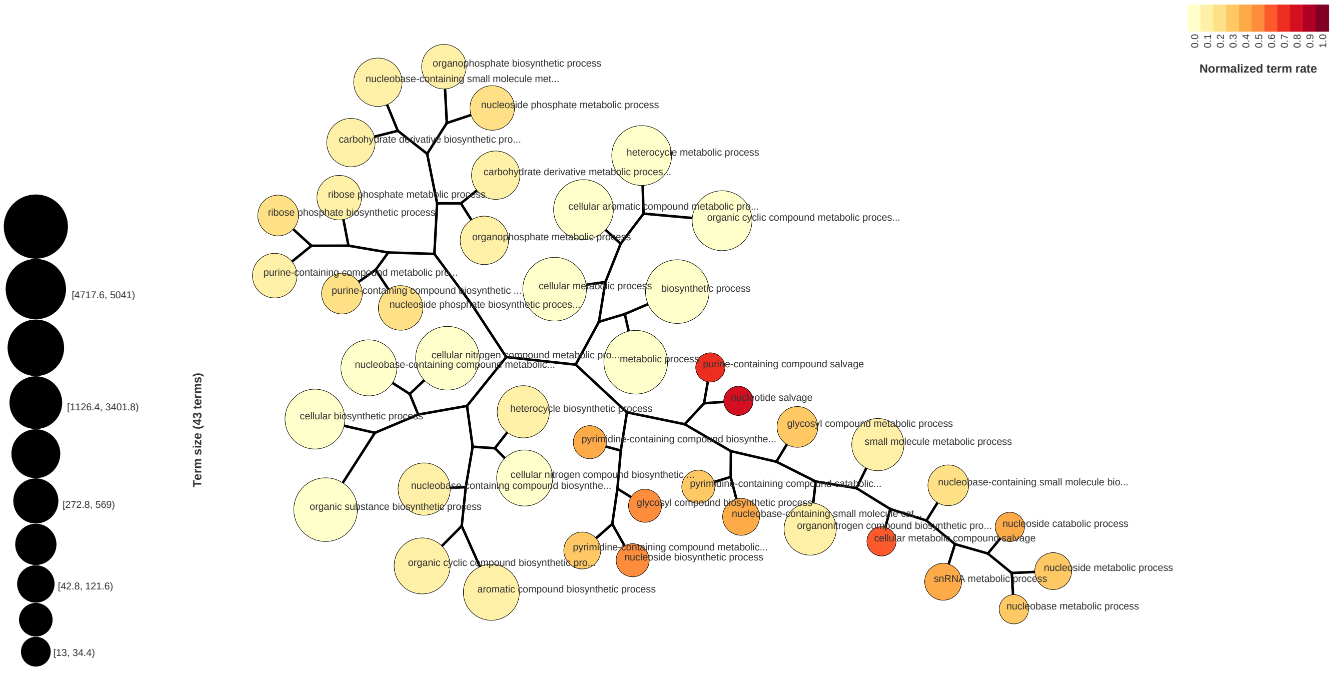

Figure S14: Complete dendrogram of cluster 8 – time-interval 1

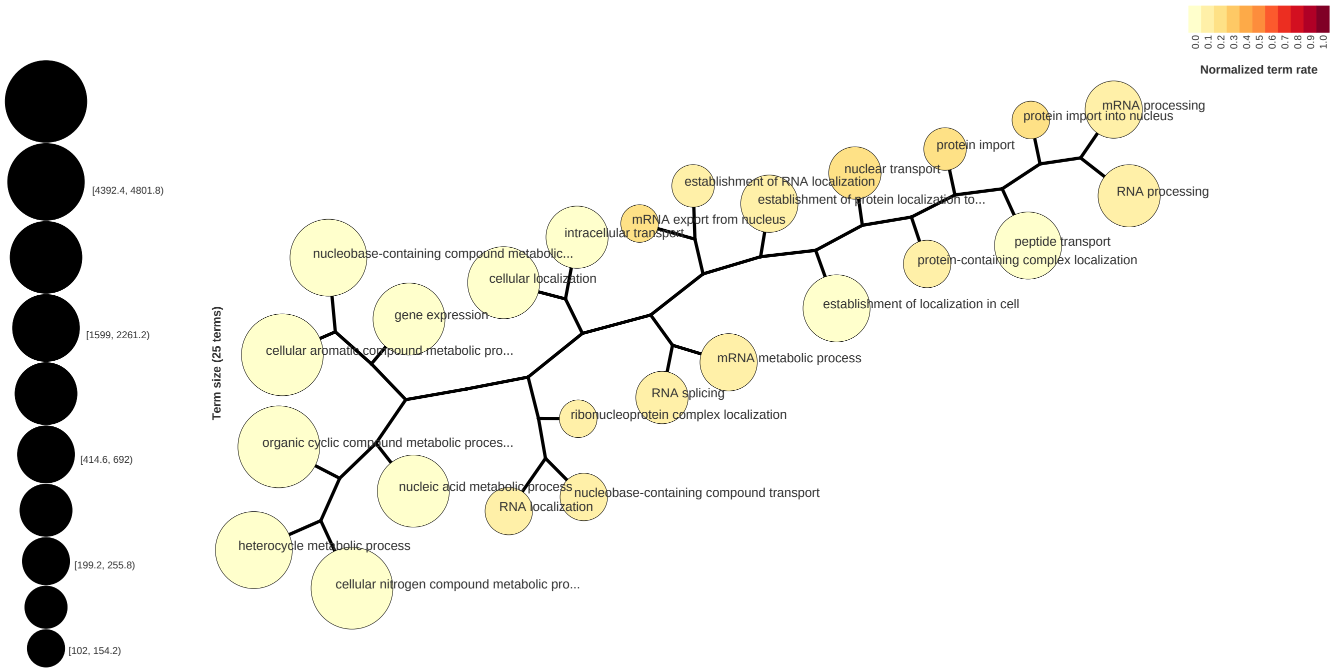

Figure S15: Complete dendrogram of cluster 9 – time-interval 1

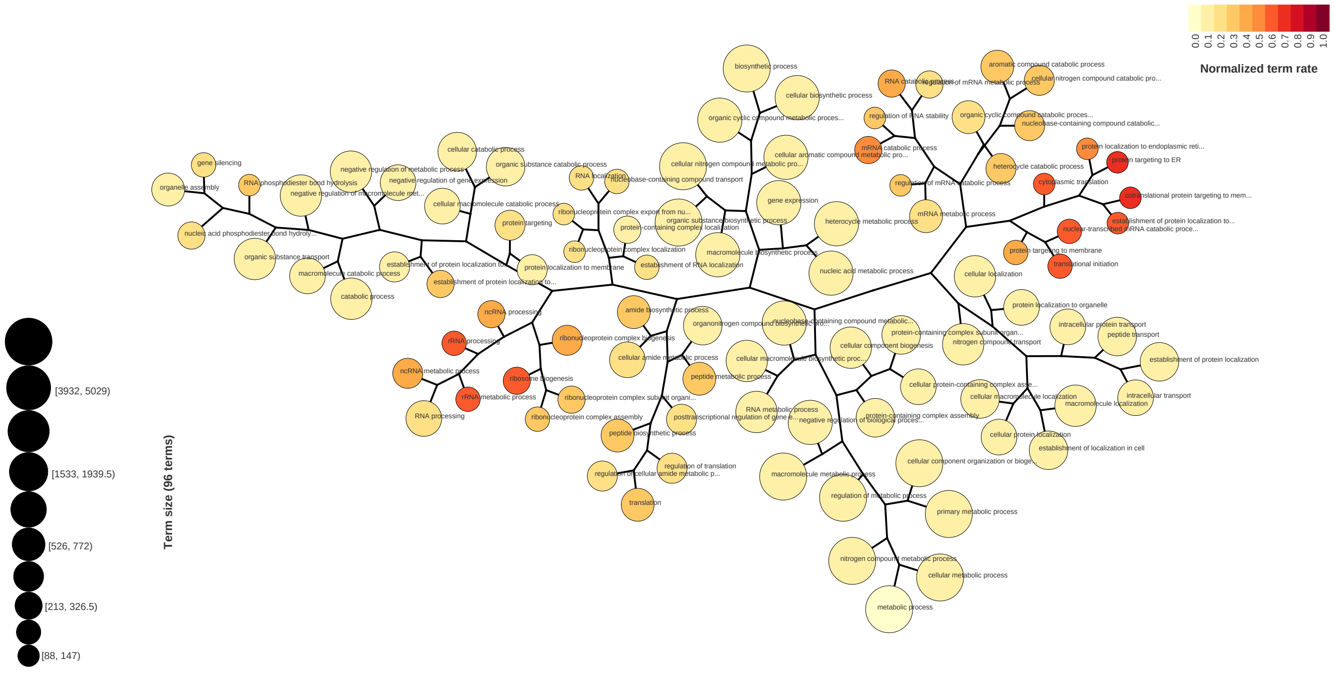

Figure S16: Complete dendrogram of cluster 10 – time-interval 1

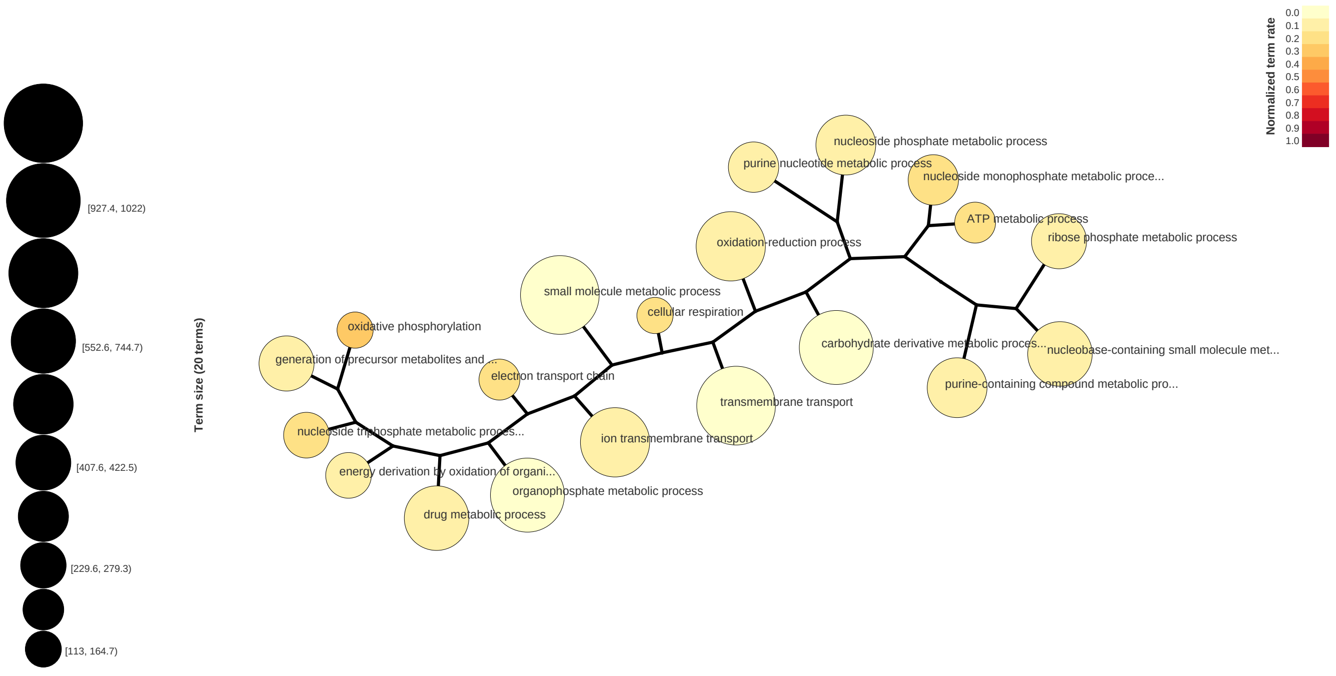

Figure S17: Complete dendrogram of cluster 11 – time-interval 1

Adjacency Matrix of Interactions Between Clusters

Time-Interval 1

Cluster	1	2	3	4	5	6	7	8	9	10	11
1	344	2	0	0	0	21	40	2	2	0	0
2	2	107	8	3	0	6	11	0	0	0	0
3	0	8	130	5	38	78	36	4	1	6	0
4	0	3	5	393	38	128	101	16	15	21	8
5	0	0	38	38	1244	1101	686	56	35	26	18
6	21	6	78	128	1101	12479	10485	490	449	794	129
7	40	11	36	101	686	10485	79128	2674	2269	1616	86
8	2	0	4	16	56	490	2674	2217	905	484	59
9	2	0	1	15	35	449	2269	905	1524	692	58
10	0	0	6	21	26	794	1616	484	692	17467	94
11	0	0	0	8	18	129	86	59	58	94	707

Time-Interval 2

Cluster	1	2	3	4	5	6	7	8	9	10	11
1	12630	4636	828	1053	102	844	54	209	127	66	2
2	4636	59802	7791	5184	470	2418	95	559	186	81	9
3	828	7791	7090	5299	342	1913	52	180	61	28	4
4	1053	5184	5299	14532	1588	5060	177	444	222	149	7
5	102	470	342	1588	925	3712	49	480	83	30	3
6	844	2418	1913	5060	3712	91802	2377	8090	454	642	56
7	54	95	52	177	49	2377	767	984	141	65	1
8	209	559	180	444	480	8090	984	43211	1492	291	26
9	127	186	61	222	83	454	141	1492	2685	1488	10
10	66	81	28	149	30	642	65	291	1488	1006	41
11	2	9	4	7	3	56	1	26	10	41	182

Figure S18: Adjacency matrix used to draw the internal and external connectivity lines of PPI networks of time-interval 1 and 2, shown in Figure 1b) and 1d), respectively

SUPPLEMENTARY TABLES S1 AND S2

Tables S1 and S2 (inside file Table1.XLSX) contain the list of enriched terms of time-interval 1 and 2 clusters, respectively, as described below:

Field	Description
**GO.ID**	Gene Ontology term ID
**Term**	Human-readable term name
**Annotated**	Number of genes annotated for a specific GO term
**Significant**	Number of differentially expressed genes identified
**Expected**	Number of differentially expressed genes expected by the topGO statistical test
**pValue**	p-values adjusted by the Benjamini-Hochberg procedure.
